# Deconvolution of bulk gene expression profiles reveals the association between immune cell polarization and the prognosis of hepatocellular carcinoma patients

**DOI:** 10.1002/cam4.6197

**Published:** 2023-06-27

**Authors:** Yen‐Jung Chiu, Chung‐En Ni, Yen‐Hua Huang

**Affiliations:** ^1^ Institute of Biomedical Informatics National Yang Ming Chiao Tung University Taipei Taiwan; ^2^ Department of Biomedical Engineering Ming Chuan University Taoyuan Taiwan; ^3^ Center for Systems and Synthetic Biology National Yang Ming Chiao Tung University Taipei Taiwan

**Keywords:** bulk gene expression profiles, CD8 T cell, deconvolution, hepatocellular carcinoma, immune cell polarization, therapy response subtypes

## Abstract

**Background:**

Many studies have utilized computational methods, including cell composition deconvolution (CCD), to correlate immune cell polarizations with the survival of cancer patients, including those with hepatocellular carcinoma (HCC). However, currently available cell deconvolution estimated (CDE) tools do not cover the wide range of immune cell changes that are known to influence tumor progression.

**Results:**

A new CCD tool, HCCImm, was designed to estimate the abundance of tumor cells and 16 immune cell types in the bulk gene expression profiles of HCC samples. HCCImm was validated using real datasets derived from human peripheral blood mononuclear cells (PBMCs) and HCC tissue samples, demonstrating that HCCImm outperforms other CCD tools. We used HCCImm to analyze the bulk RNA‐seq datasets of The Cancer Genome Atlas (TCGA)‐liver hepatocellular carcinoma (LIHC) samples. We found that the proportions of memory CD8^+^ T cells and Tregs were negatively associated with patient overall survival (OS). Furthermore, the proportion of naïve CD8^+^ T cells was positively associated with patient OS. In addition, the TCGA‐LIHC samples with a high tumor mutational burden had a significantly high abundance of nonmacrophage leukocytes.

**Conclusions:**

HCCImm was equipped with a new set of reference gene expression profiles that allowed for a more robust analysis of HCC patient expression data. The source code is provided at https://github.com/holiday01/HCCImm.

## Graphical abstract

The HCCImm model, a cell composition deconvolution tool, was developed to accurately estimate the abundance of 17 cell types within the microenvironment of hepatocellular carcinoma (HCC). The differentially expressed genes (DEGs) were used to construct an HCC reference gene expression signature matrix. Subsequently, predicted abundance of HCC cells were excluded, and the proportions of immune cell types were normalized. Survival analysis was performed to investigate the relationship between immune cell abundance and patient prognosis. This comprehensive workflow enables a detailed exploration of gene expression patterns and their impact on patient outcomes. Notably, the abundance of CD8 T cells has been identified as a significant factor associated with prognosis and the effectiveness of drug treatments.

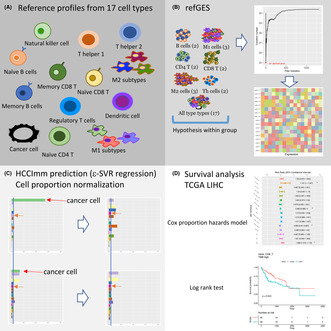

## 1 INTRODUCTION

Primary liver cancer is the seventh most common cancer type and the second most common cause of cancer‐related mortality in the world,^1^ and hepatocellular carcinoma (HCC) accounts for nearly three‐quarters of all liver cancer cases and is the dominant type of liver cancer.^1^ Treatment of advanced HCC is challenging, and systemic chemotherapy has toxic side effects and no survival benefits.^2^, ^3^ Recently, immunotherapy, in particular immune checkpoint inhibition (ICI), has been used as a clinical treatment for HCC. By blocking the cancer cell‐mediated suppression of immune cells, they can be reactivated and kill cancer cells. ICI has been approved as a first‐line treatment for advanced HCC cases^4^; however, only a subset of patients seem to benefit from ICI therapy. It has been hypothesized that the poor ICI response rate might be related to individual differences in the types and relative abundances of immune cells in the tumor microenvironment (TME).^5^ In addition, Wang and Li suggest that ICIs might not be a particularly favorable method to treat HCC because immune checkpoint genes are not highly expressed in LIHC patients that have a high tumor mutational burden (TMB),^6^ which suggests that the characteristics of the HCC TME are different from those of other cancers.

The TME is a complex ecosystem that consists of a range of different types of cells; these different cell types include not only cancer cells but also numerous interstitial cells and infiltrating immune cells. The abundance and density of these tumor‐infiltrating immune cells (TIICs)^7^ may be associated with patient survival and cancer treatment efficacy. TIICs seem to interact with cancer cells or other noncancer interstitial cells in ways that are not yet fully understood.^8^ Immune cells might be able to modulate cancer progression by regulating the growth and metastasis of tumor cells. There are also likely to be interactions between immune cells via cytokines or surface proteins, which creates a complex network among immune cells.^9^, ^10^

Immune cells are regulated by a variety of cytokines as well as by cell–cell contacts, which results in their polarization to perform a number of distinct functions. The immune cells present in a given sample may have been polarized in a number of different directions, and these complex changes impose unexpected influences on the prognosis of cancer patients. In recent years, many studies have found that different polarizations of the same immune cell type may have diverse effects on cancer cells. Some polarized forms are able to inhibit the growth of cancer cells or even kill them, while others are able to promote the growth and/or metastasis of cancer cells.^11^ For example, macrophage cells can be polarized into classically activated macrophages (M1), or alternatively, they can become alternatively activated macrophages (M2). M2 cells promote the growth of cancer cells, while M1 cells inhibit the differentiation of cancer cells.^12^ A few studies have reported that a high M1/M2 ratio is associated with greater survival of cancer patients.^13^, ^14^ Furthermore, M2 cells can be further divided into four subtypes, namely, M2a, M2b, M2c, and M2d, each of which has been suggested to exert different effects within the TME. Tumor‐associated macrophages (TAMs), which have an immunosuppressive role and protumor properties, are referred to as M2d cells.^15^, ^16^

In addition, CD8^+^ T cells and their functional subsets have been highlighted in many studies due to their roles in cancer immunotherapy.^17^ A higher density and a greater abundance of CD8^+^ T cells in the TME have been associated with a favorable prognosis for cancer patients^18^; however, in the TME of HCC patients, resident memory CD8^+^ T cells may differentiate into exhausted phenotypes, and a higher abundance of such exhausted memory CD8^+^ T cells has been associated with the poor prognosis of HCC patients.^19^ In contrast, the activated CD8^+^ T cells differentiated from naïve CD8^+^ T cells, compared to those effector cells derived from memory CD8^+^ T cells, exhibit a better resistance to tumor‐induced immune suppression and reveal a more potent tumor‐specific cytotoxic activity.^20^

Exploring which polarized immune cells are significantly associated with cancer patient prognosis is likely to allow new insights into the application and development of immunotherapy. A useful approach is to investigate the immune cell composition of publicly available tumor sample datasets. Although experimental methods such as flow cytometry and immunohistochemistry staining (IHC) are able to determine the abundance of immune cell types, such information is usually unavailable in public‐domain datasets.^21^ Therefore, many cell composition deconvolution (CCD) methods have been developed to infer the immune cell admixture based on the bulk gene expression profile (bulk GEP) data of tissues. These CCD methods include linear least‐square regression,^22^, ^23^ nonnegative matrix factorization,^24^, ^25^, ^26^ quadratic programming,^27^, ^28^ and 𝜈‐support vector regression.^29^ Usually, when using a deconvolution method, a set of cell‐specific gene expression profiles have been curated and used as the reference (reference GEP), and this reference GEP is then used to predict the relative abundance of different immune cells from the bulk GEP of a given new cancer sample. Among these computational deconvolution approaches, CIBERSORTx developed by Newman et al. is one of the most widely used methods to predict the immune cell composition of a bulk GEP derived from a tumor sample.^29^, ^30^ Moreover, marker gene‐based approaches, such as single sample Gene Set Enrichment Analysis (ssGSEA), are able to evaluate the expression values of the genes in a sample. These are characteristic for each of the selected cell types and therefore facilitate individual abundance estimations.^21^

When different cell type quantification methods are applied to analyze the bulk gene expression profiles of cancer patient tissue samples, discrepancies in the results can occur when the predicted associations of immune cells with HCC patient survival are explored. For example, regulatory T cells (Tregs) are an immunosuppressive subtype of CD4^+^ T cells and are known to promote tumor growth. Tu. analyzed ICOS^+^ FOXP3^+^ Tregs in 57 HCC patient tissue samples by immunohistochemistry (IHC) and reported that the infiltration of ICOS^+^ FOXP3^+^ Tregs showed a negative correlation with patient survival.^31^ In contrast to this finding, Foerster et al. analyzed the TCGA‐LIHC dataset using a marker gene‐based approach and found that a higher abundance of Tregs was positively associated with patient survival.^32^ Finally, Peng et al. analyzed the same dataset with CIBERSORT and reported that the abundance of Tregs was significantly higher in HCC patients with poor prognosis.^33^

These discrepancies among the results of previous studies seem to reflect the fact that some of the approaches used have a lower level of performance. According to benchmarks created from synthetic bulk gene expression profile datasets, deconvolution‐based approaches usually outperform marker gene‐based methods^21^; however, it can be clearly argued that further study in this area is needed. When CIBERSORT was applied to predict immune cell compositions using cancer patient datasets, a few limitations were noticeable. For example, when using CIBERSORT to analyze the TCGA‐LIHC dataset, in as many as 80% of the cases, the cell composition predictions were unable to pass the Monte Carlo test (*p‐*value >0.05).^32^ This finding suggests that when using CIBERSORT, the association between cell admixture and patient survival might be biased because only a small subset of LIHC patients was assessed. In addition, when deconvolution‐based methods are used, Sturm et al. noticed that nonspecific signature genes can cause high background predictions, or “spillover,” for certain cell types.^21^ As cancer cells are highly likely to make up the largest cell group in a tumor and thus the highest proportion in the TME, we believe that there is a concern that, without specifically including cancer cells in the reference GEP, a deconvolution‐based approach might give rise to additional spillover due to the gene expression signals contributed by the cancer cells in the sample.

Exploring the roles of infiltrating lymphocytes and other immune cells in the TME is a popular research topic. Available evidence supports the hypothesis that the cell heterogeneity present in the TME seems to have an impact on cancer patient survival and response to therapy.^34^ A better understanding of the complexity present within the TME is likely to provide further insights that will allow the development of new HCC therapies. Based on recent studies of human HCC samples using single‐cell RNA‐seq,^35^, ^36^ there is high cell heterogeneity in TME of liver cancer, containing a diversity of immune cell types, including dendritic cells (DCs), natural killer (NK) cells, CD4^+^ T cells, CD8^+^ T cells, and TAMs. Zhang et al. also found that Treg and exhausted CD8^+^ T cells were enriched in human HCC tumor tissues and suggested that it is necessary to clarify their association with HCC patient survival.^35^

CCD methods may have limitations due to spillover from the overlapping gene expression profiles among different cell types.^21^ Various CCD tools, including CIBERSORTx,^30^ EPIC,^37^ TIMER,^38^ Dtangle,^39^ TumorDecon,^40^ and SCDC,^41^ have been developed to predict the abundance of immune cells. For example, CIBERSORTx can predict 22 types of immune cells. EPIC can estimate the abundance of eight cell types, including immune cells and cancer‐associated fibroblasts. TIMER provides immune cell estimations for diverse cancer types. Dtangle can estimate 11 types of immune cells in Lyme disease. TumorDecon can predict 12 types of immune cells for quantitative pharmacology models. SCDC uses an ENSEMBLE approach to develop CCD algorithms. However, despite the improved capabilities of these tools, spillover from the gene expression profiles of HCC cancer cells remains a challenge for CCD methods.

Our tool, HCC Immune Estimating (HCCImm), has been explicitly designed to analyze the immune TME in liver cancer by a unique reference gene expression signature matrix (refGES) built from a liver cancer cell line to improve the accuracy of cell type deconvolution. As a result, HCCImm can more accurately distinguish between immune cells and liver cells than other CCD methods.

Therefore, this study aims not only to create a new CCD method that can be applied to analyze the TCGA‐LIHC dataset but also to more diversely quantify the polarized populations of immune cells. The inferred immune cell compositions could be associated with HCC patient survival. To this end, we built our reference gene expression profile by including a greater range of immune cell types. For example, we have included more differentially polarized forms of macrophages than just the binary categorization into M1 and M2. We also expanded the types of CD8^+^ T cells included and further divided them into two subtypes, namely, naïve and memory CD8^+^ T cells, as each has specific immune characteristics. Cytotoxic T cells expressing the CD8 cell‐surface marker are the most powerful effectors associated with the cancer immune response,^42^ but in the TME, tissue‐resident memory CD8^+^ T cells may express various dysfunctional markers, such as PDCD1 and CTLA4^43^; these markers have also been included. As a comparison, CCD methods such as CIBERSORTx and quanTIseq can predict only a single fraction value for the CD8^+^ T‐cell type.^29^, ^30^, ^44^

## 2 STUDY DESIGN

**FIGURE 1:**
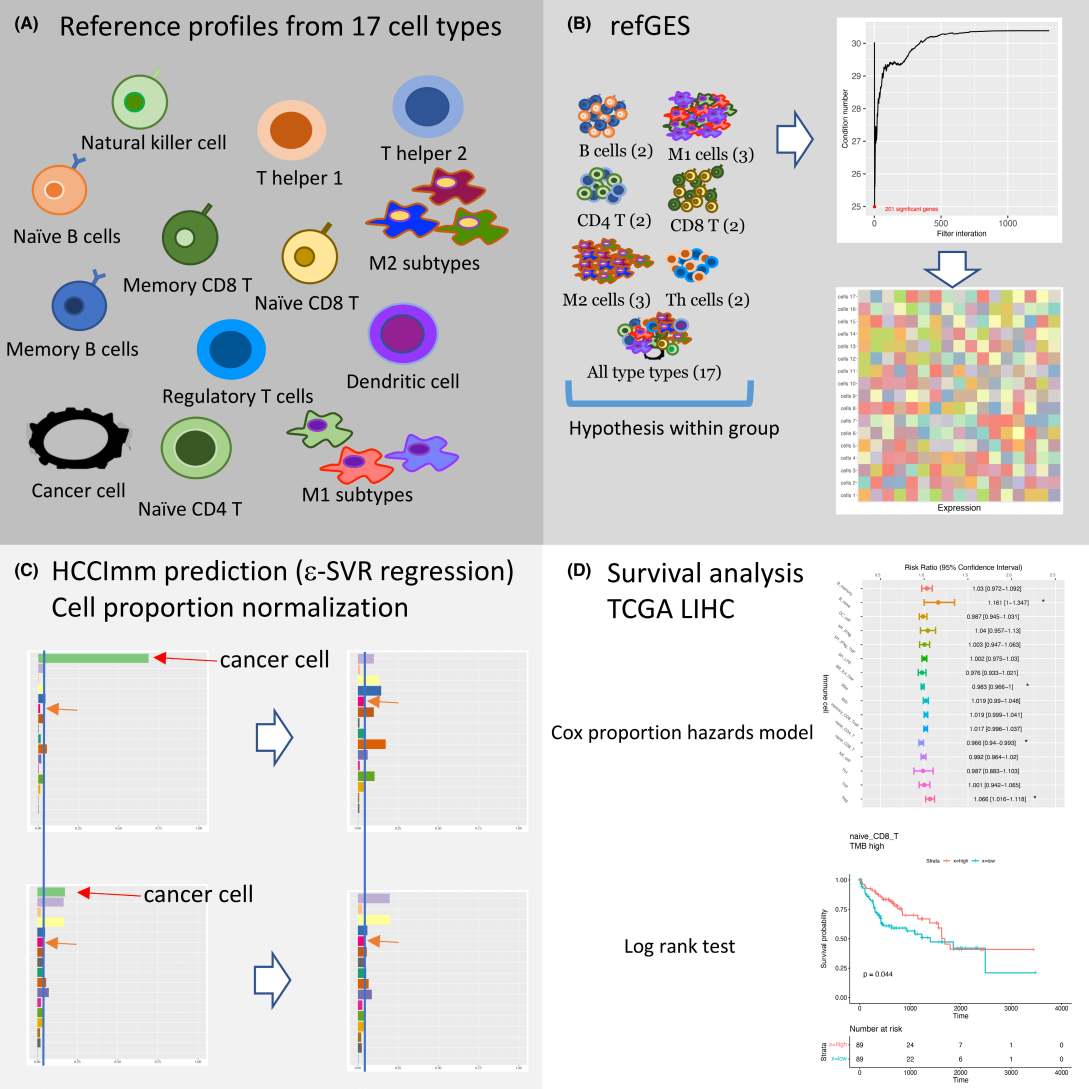
The workflow of this study (A) The gene expression profiles of 17 cell types were downloaded from NCBI GEO. (B) Creating refGES for 17 types of cells. The differentially expressed genes (DEGs) within each of the seven groups were ranked by *p*‐value, and then minimization of the condition number was performed to select the top DEGs. (C) HCCImm was built using ε‐SVR regression. After removing the predicted fraction of HCC cells, the fractions of the remaining 16 types of immune cells were normalized to sum 100%. (D) Survival analysis was performed to determine whether there was an association between the predicted abundance of immune cell types and patient prognosis.

The workflow overview of this study is presented in Figure 1. To construct the reference gene signature matrix, which was used for regression of the immune cell types in HCC samples, we collected the gene expression profiles of 17 distinct types of cells (Figure 1A). These cell types were an HCC cell line, memory B cell, naïve B cells, DC cells, M1 cells induced by interferon γ (IFN γ), M1 cells induced by IFN γ, and tumor necrosis factor (TNF), M1 cells induced by lipopolysaccharide (LPS), M2 cells induced by IL4 and dexamethasone (DEX), M2a cells induced by IL13 or IL4, M2c cells induced by IL10, NK cells, naïve CD8^+^ T cells, memory CD8^+^ T cells, naïve CD4^+^ T cells, T helper 1 cells (Th1 cells), T helper 2 cells (Th2 cells), and Tregs (Table 1). The different macrophage polarization forms in the gene expression profiles are labeled according to the corresponding cytokines used for their in vitro induction.^45^

**TABLE 1:** Cell types and replicates used to build the reference gene expression signature matrix.

Cell type	Replicates	Cell type	Replicates
HCC_cell_lines	19	B_memory	18
M1_IFNγ	9	B_naive	25
M1_IFNγ_TNF	4	Memory_CD8_Tcell	12
M1_LPS	6	Naïve_CD8_T	15
M2_IL4_Dex	15	Naïve_CD4_T	10
M2a	12	Treg	10
M2c	8	Th1	7
DC_cell	13	Th2	9
NK_cell	12		

To create the refGES for CCD, one idea might be to perform ANOVA and t‐test analysis to identify the differentially expressed genes across the 17 cell types. However, within the 17 cell types, there are closely related subsets, and each cell‐type subset is likely to have highly correlated expression profiles. For example, the three types of M1 cells induced by different cytokines, as listed in Table S1, have very similar gene expression profiles, as revealed by cluster analysis. If the cell‐specific signature genes were collected by investigating the differentially expressed genes (DEGs) in the 17 cell types via ANOVA, it is possible that the acquired cell signature genes might not be sufficient to distinguish these highly correlated cell types. In addition, highly correlated independent variables can cause multicollinearity during linear regression. To mitigate these concerns, we created our refGES by specifically choosing genes that are differentially expressed between the closely related cell subtypes (Figure 1B).

For the implementation of the computational code for HCCImm, we used the linearSVR program in the Python package “scikit‐learn.” We chose ε‐support vector regression (ε‐SVR), subject to an *L1* penalty, as the core algorithm to perform the deconvolution in this study. Our ε‐SVR‐based deconvolution method, HCCImm, was designed in‐house to analyze bulk expression profiles from samples with an unknown cell composition to predict the proportions of the 16 immune cell types present in liver cancer tissue samples.

## 3 IMPLEMENTATION

### 3.1 The refGES

The aim of this study was to quantify the 16 immune cell types described above, as well as HCC cells, present in the TME of HCC, and, therefore, 196 microarray samples were downloaded from NCBI GEO, and at least four replicate samples were included for each of the 17 cell types (Figure 1A). All of the samples downloaded from NCBI GEO were analyzed using the same platform, Affymetrix U133 plus 2 (NCBI GEO platform accession: GPL570). The numbers of replicates for the different types of cells are listed in Table 1, and the NCBI GEO sample accession numbers for those microarray samples are provided in Table S1.

The microarray data were quantile normalized using the Robust Multiarray Average (RMA) procedure offered in the R package “affy”.^46^ ANOVA was performed using the R package “statistics” to identify the genes that had significantly higher expression in certain types of immune cells compared to that in other cell types. The expression values of those differentially expressed genes (DEGs) were used to build the initial matrix for the gene expression signatures of the immune cells.

Our approach to construct the refGES consisted of three steps (Figure 1B). First, in addition to the 17 cell‐type group, we further divided the 17 cell types into six groups. Each of the six immune cell groups consisted of the cell types that had closely correlated gene expression profiles (Table S1). For example, the M1 group consisted of M1_LPS, M1_IFNγ, and M1_IFNγ_TNF, while the M2 group consisted of M2a, M2_IL4_DEX, and M2c.

Second, to construct the refGES, differentially expressed genes were selected from each of the seven cell groups, including the 17‐cell‐type group and the six groups of immune cell subsets. In each of the seven groups, all of the genes were sorted by their *p‐*values in ascending order. The top *k* differentially expressed genes were collected from each group.

Third, the median expression values of these selected differentially expressed genes from each of the 17 cell types were used to build the initial refGES, with the dimension of approximately *k* genes × 7 groups × 17 cell types × iteration #. We set *k* = 16, but other *k* values might work equally as well. A differentially expressed gene in one of the seven groups might be found to be differentially expressed again in one or more of the six groups, and therefore, in such circumstances, the number of genes to be included to build the refGES could be less than *k* in each iteration for each of the seven cell type groups. Next, we used condition numbers to determine how many differentially expressed genes should be included in the refGES. Next, we used QIAGEN's Ingenuity^®^ Pathway Analysis (IPA^®^, QIAGEN) to identify the diseases and functions associated with these genes (Supplementary Figure S1).

To make the refGES more robust against input variations or noise, we used condition numbers as a means to determine how many differentially expressed genes should be included in the final matrix. Technically, the condition number of a matrix is the product of the norm of the matrix and the norm of its inverse. It is a measurement of how sensitive a mathematical function might be to changes or errors in the input data. In numerical analysis, a function with a low condition number is considered to be well‐conditioned and likely to be a relatively stable solution when there are small fluctuations in the input data. A number of CCD methods have applied the minimization of condition numbers to optimize the refGES.^22^, ^27^, ^29^ Specifically, the R function “kappa” was used to estimate the condition number of each matrix. Finally, we created the refGES matrix that consisted of the median gene expression values of 201 genes expressed in the 17 types of cells, including 16 immune cell types, as well as HCC cells (Table S1).

Each of the cell types listed in Table 1 can be further categorized into distinct subsets. However, these subsets were not inputted as immune cell subtypes when building our refGES. For example, in the reference microarray dataset of memory CD8^+^ T cells, three subsets were noted, including central memory CD8^+^ T cells, effector memory CD8^+^ T cells, and CD45RO^−^ memory CD8^+^ T cells. However, when building our refGES, all three memory CD8^+^ T subsets of microarray data were regarded as a single type of memory CD8^+^ T cells. We chose to use this kind of approach because we noticed that the gene expression patterns of these subsets belonging to the same cell types bear high resemblance. When using the refGES consisting of additional immune cell types corresponding to these distinct subsets, the regression model made poor predictions in benchmark testing using the PBMC samples or the samples containing only pure cell types, deviating immensely from the true proportions.

Perhaps a critical reason to prevent us from further dividing the cell subsets to include more cell subtypes in our method is due to the resolution limits of expression signal deconvolution. One challenge in developing CCD methods is the high correlation of the expression profiles of closely related cell types.^47^ The gene expression signals derived from low‐abundance cell types might be masked by the expression of the same gene(s) in a more abundant cell subset.^9^

Moreover, a remaining issue is whether the constructed refGES can be applied to estimate the cell compositions in samples in which there are certain subsets of immune cells that do not align perfectly with the reference cell types in our refGES. For example, it is unclear whether the predicted fraction of memory CD8^+^ T cells reflects the true compositions in samples containing distinct subsets of memory CD8^+^ T cells. Such subsets of memory CD8^+^ T cells are referred to as nontypical memory CD8^+^ T (NT‐MCD8T) cells in the following text to distinguish them from the reference memory CD8^+^ T cells samples used to build our refGES for the HCCImm method. Therefore, to address this issue, at least partially, an assessment was carried out by using two additional datasets, NCBI GEO GSE85074 and GSE26495, in which there are microarray data of memory CD8^+^ T cells that are either PD1^high^, PD1^low^, effector, or central subtype (Table S2). This assessment revealed that the gene expression profiles of these subsets of memory CD8^+^ T cells are indeed more similar to those of memory CD8^+^ cells than to those of naïve CD8^+^ T cells in our reference datasets.

To estimate the HCC cell fraction in the HCC samples, we decided to use the gene expression profiles of Huh7 cells to build our refGES. An important reason is that the GEP of the Huh7 cell line, which is a human hepatoma‐derived cancer cell line, mimics that of human HCC‐derived metabolic genes.^48^ To further assess whether Huh7 might be superior to other HCC cell lines as a representative of HCC tumor cells, we performed a correlation analysis to determine the correlation of Huh7 and HepG2 cell lines with human HCC samples. The NCBI GEO accession numbers of these microarray data, including 10 Huh7, 9 HepG2, and 50 human HCC samples, are listed in Table S3. The GEPs of liver tumor samples derived from 50 HCC patients (NCBI GEO GSE45267) were more similar to those of Huh7 cells than to those of HepG2 cells (Supplementary Figure S2).

For CCD method benchmarking, it is essential to map the cell types of the different methods to the same cell‐type ontology. For example, CIBERSORTx is designed to estimate the fractions of 22 cell types in a cell admixture,^29^, ^30^ whereas quanTIseq can quantify only 10 immune cell types.^44^ Since different methods elucidate cell types in more or less detail, it is a common practice when assessing CCD methods such as CIBERSORTx to sum up the predicted fractions for the cell subsets belonging to the same cell type. Conversely, when CCD methods can give only a single fraction value for one cell type but not the detailed fractions for distinct cell subsets belonging to that one cell type, this predicted fraction corresponds to the sum of the cell subset fractions.^44^

### 3.2 ε‐support vector regression

One common CCD approach is the use of linear models, where the gene expression levels of a mixed sample (*x*) are modeled as a linear combination of the gene expression levels of the individual cell types that make up the sample. In this context, bulk GEP (*b)* refers to the gene expression profile of the mixed sample, while refGES (*a*) refers to the reference gene expression profiles of the individual cell types. The linear model is used to calculate the proportions of each cell type in the mixed sample by solving a system of linear equations.

Deconvolution can be conceived as finding the solution to the convolution equation:

$$b_{i}=a_{i,1}x_{1}+a_{i,2}x_{2}\ldots a_{i,j}x_{j}$$

where *b*_*i*_ is the expression level of gene *i* in a sample of the cell mixture, *a*_*i,j*_ is the expression level of gene *i* in cell type *j* (derived from the reference expression signature for this cell type), and *x*_*j*_ is the unknown proportion of cell type *j* in the cell mixture. Among the available CCD tools, CIBERSORTx uses a 𝜈‐support vector regression (SVR)‐based approach, and this tool has outperformed other tools in benchmarking experiments.^29^ Since SVR seems to be superior to the other regression methods in making predictions, we chose one type of SVR, ε‐insensitive support vector regression (ε‐SVR) which can provide a sparse solution, as the core algorithm to perform deconvolution in this study. Our ε‐SVR‐based method is named HCCImm. To implement HCCImm, we used the linearSVR function in the Python package “scikit‐learn.” Unlike the 𝜈‐SVR used by CIBERSORTx, ε‐SVR does not control the proportion of the support vectors to be used in the final model.^49^ As a result, an ε‐SVR‐based deconvolution model might have higher flexibility when combining different predictor variables without setting a lower bound for the support vectors. After all, it would be arbitrary to decide a lower bound for the immune cell types of one bulk tissue without prior knowledge of its cell composition. In addition, the *L1*‐loss function was applied to minimize the mean average error (MAE) between the predicted and real values.^50^ Compared to the *L2*‐loss function, the *L1*‐loss function makes the regression model more robust to outlier values in the input data. The *L2*‐loss function can produce much larger errors for outliers because this approach takes into consideration the squared differences.

### 3.3 Other linear regression models

In addition to ε‐SVR, we tested a number of other linear regression models, each being subject to one of the three types of regularization. The objective function and the loss terms are presented below.

$$\text{Objective function}:\min\frac{100}{i}\sum_{n=1}^{i}\left|\frac{b_{n}-\hat{b_{n}}}{b_{n}}\right|+\text{regularized term},\text{where}$$

$$\hat{b}=a_{i,1}x_{1}+a_{i,2}x_{2}\ldots a_{i,j}x_{j}$$

$$L1\;\text{loss}:\alpha \sum \left|x\right|$$

$$L2\;\text{loss}:\alpha \sum x^{2}$$

$$\text{Elastic}\;\mathrm{Net}\;\text{loss}:\alpha \sum \left|x\right|+\beta \sum x^{2}$$

Subject tox≥0

### 3.4 Benchmarking of cell deconvolution methods

#### 3.4.1 Analysis of pure cell types

The benchmarking test to assess the performance of HCCImm initially used pure cell types and followed a leave‐one‐out strategy. In each test run, one of the 196 microarray samples obtained from NCBI GEO to build the refGES for the seventeen cell types was used as the test dataset for deconvolution, while the remaining 203 samples were used to build the refGES for testing using the aforementioned ANOVA‐based procedure. Bar plots were produced to show the distributions of the predicted cell‐type fractions in those pure‐cell samples. The results were compared with the predictions made by CIBERSORTx and quanTIseq, as well as the least absolute shrinkage and selection operator (LASSO), *L1*‐, *L2*‐, and elastic net‐regularized linear regressions.

#### 3.4.2 Analysis of memory CD8 ^+^ T‐cell sample subsets by HCCImm

Memory CD8^+^ T cells have many subsets, including central memory CD8^+^ T cells, effector memory CD8^+^ T cells, and others, such as PD1^+^ CD8^+^ T cells. To confirm which memory CD8^+^ T‐cell subsets HCCImm could estimate, two additional CD8^+^ T‐cell datasets, GSE85074 and GSE26495, were assessed using the model.

#### 3.4.3 Analysis of human PBMCs by flow cytometry

The performance of HCCImm was also assessed by using real mixtures of gene expression profiles derived from PBMCs. First, a set of microarray samples of human PBMCs, GSE107990, was downloaded from NCBI GEO; this dataset has 164 samples. Since each of these PBMC samples had its immune cell composition determined by flow cytometry, we compared the cell abundance predicted by HCCImm with the experimentally determined composition. The platform used to obtain those microarray samples was the Illumina HumanHT‐12 V4.0 expression beadchip and the raw dataset was quantile‐normalized using the R package “preprocessCore.” We followed a simple cross‐platform quantile normalization approach that is described in a previous study.^51^ Mean absolute errors (MAEs) and Pearson's correlation coefficients were calculated to compare the performances of HCCImm against those of other CCD tools.

Next, to assess whether HCCImm can be applied to analyze the cell composition of RNA‐seq datasets, such as TCGA cancer samples, a set of nine RNA‐seq samples derived from the human PBMC dataset GSE107572 was downloaded from NCBI GEO; these samples had their composition confirmed by flow cytometry for eight types of immune cells.^44^ To perform this benchmark test, the raw counts of the RNA‐seq data were transformed by transcripts‐per‐millions (TPM) normalization. Following this, the normalized values were further adjusted to make the RNA‐seq data have a distribution with a maximum value close to that of the microarray datasets used to build our refGES. When using CIBERSORTx and quanTIseq, gene expression values in TPM were used, as has been suggested by two papers.^44^, ^52^ The cell type mapping from the predictions made by CIBERSORTx, quanTIseq, and HCCImm is provided in Table S4(A).

A common approach for evaluating the performance of CCD tools involves mapping the predicted fractions of immune cell subtypes to higher‐level immune cell types.^21^, ^34^ This strategy typically starts by comparing the predicted fractions of immune cell subtypes with true experimentally obtained fractions. In cases where the true fraction is only available for a higher‐level immune cell type, but not for its constituent subtypes, the predicted fractions of the subtypes are usually aggregated to estimate the fraction of the higher‐level immune cell type.

As an example, HCCImm can predict the fractions of two subtypes of CD8^+^ T cells: naïve CD8^+^ T cells and memory CD8^+^ T cells. In the evaluation using the GSE107990 dataset, the predicted fractions for these subtypes were summed to obtain an aggregated fraction value for the overall CD8^+^ T‐cell population (Table S4A).

Other CCD tools have been assessed in a similar manner, such as CIBERSORT, CIBERSORTx, and quanTIseq.

#### 3.4.4 Analysis of the simulated bulk gene expression profiles of liver cancer tissue samples

To evaluate the ability of HCCImm to accurately estimate the cell composition of the highly cancerous HCC TME, we conducted in silico simulations by spiking the expression signals of 16 immune cell types into the microarray data of an HCC cell line. We simulated bulk gene expression profiles of liver cancer tissue by randomly selecting one HCC cell sample from five datasets (GEO GSE68927, GSE112788, GSE128517, GSE75024, or GSE78736). Then the expression signals for the 16 immune cell types were randomly sampled from the 185 immune cell microarray samples, as shown in Table S1.

$$\begin{array}{c} A\;\text{simulated}\;\mathrm{HCC}\;\text{gene expression profile}=P_{\mathrm{HCC}}*\mathrm{HCC}\;\mathrm{GEP}\\ +\sum P_{j}*{\mathrm{GEP}}_{j},\;\text{where}\;P_{\mathrm{HCC}}+\sum P_{j}=1 \end{array}$$

*j* represents the immune cell type. Each simulated profile was generated by summing the GEPs of 17 cell types and weighting them according to their respective proportions which were randomly assigned.

Specifically, we generated 100 simulated HCC tissue profiles for each of the six different cancer‐cell fractions (*P*_*HCC*_) ‐ 15%, 30%, 45%, 60%, 75%, and 90%. Therefore, in each set of 100 simulated profiles, each profile had a predetermined *P*_*HCC*_ fraction of gene expression signals derived from a randomly selected HCC cell microarray sample, and the remaining fraction (100% ‐ *P*_*HCC*_) was composed of expression signals of 16 immune cell microarray samples which were randomly selected and weighted.

In each test run, deconvolution was performed by taking the remaining microarray samples of HCC cells and immune cells (excluding the one HCC cell sample and the 16 immune cell samples used to simulate this bulk profile with spike‐ins) to build the refGES that was to be used for testing. When assessing the predictions, a normalization was performed such that the fraction making up the HCC cell line was discarded (Figure 1C), and the fractions of the 16 types of immune cells were summed to 100%.

To investigate the agreement of the predictions made by the various CCD methods, including ours, Pearson's correlation coefficient and absolute error (AE) were calculated. Boxplots were produced to show the distributions of AEs, and the results were compared with the AEs of the predictions made by other methods. The cell type mapping to the ground truths from the predictions made by CIBERSORTx, quanTIseq, and HCCImm is provided in Table S4(B,C).

### 3.5 Analysis of TCGA‐LIHC samples

To predict the cell composition of HCC samples and to perform survival analysis of the patients, 371 RNA‐seq samples from the TCGA‐LIHC dataset^53^ were downloaded from the NCI Genomic Data Commons (NCI GDC, https://gdc.cancer.gov/) (Figure 1C,D). In addition, the tumor mutation data of the TCGA‐LIHC samples were obtained from the MC3 project (https://gdc.cancer.gov/about‐data/publications/mc3‐2017).^54^ Finally, the drug response data of the TCGA‐LIHC patients, curated by Ding et al., were downloaded from http://lifeome.net/supp/drug_response/.^55^

## 4 RESULTS

### 4.1 Building the refGES

Using the approaches mentioned in the Methods section, including prioritizing the DEGs in the cell subsets, and using the condition number calculation, a set of 201 DEGs was selected to build our refGES (Figure 1B, Supplementary Figure S3). Thus, the dimension of the final refGES matrix used in this study was 201 genes × 17 cell types. The refGES matrix is provided in Table S5.

**FIGURE 2:**
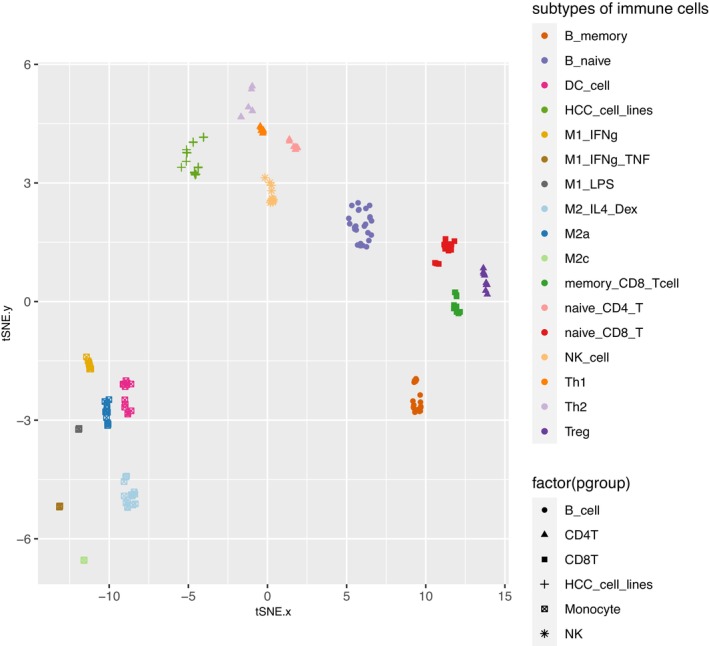
The t‐SNE plot of the 196 microarray samples using the cell type signature genes (SG) as the sample features. Each data point represents one sample and is color‐coded according to its cell type.

These 201 genes as a whole are described as the signature genes (SGs) in the following text. To reveal the clustering of the 196 microarray samples, these SGs were used to generate the t‐SNE (t‐distributed stochastic neighbor embedding) plot,^56^ and from this plot, it is clear that the samples form a number of distinct clusters, each corresponding to a single cell type (Figure 2). In contrast, when using the t‐SNE plot generated with all of the ~20,000 gene expression values as the sample features, some clusters consisted of samples that belong to different cell types (Supplementary Figure S4). For example, clusters made up of heterogeneous cell types were found for the DC samples, the M2c samples, the M1_LPS samples, and the M2a samples. Furthermore, memory CD8^+^ T‐cell samples and naïve CD8^+^ T‐cell samples overlapped. Finally, the NK, Th1, Th2, and naïve CD4^+^ T‐cell samples did not form clear‐cut clusters (Supplementary Figure S4). This result suggests that the expression values of these SGs can be used to distinguish the 17 types of cells that make up the samples.

We performed pathway enrichment analysis to explore the functions of the SGs and found that half of them were implicated in four types of functions/diseases involving the liver, namely, liver damage, necrosis of the liver, inflammation of the liver, and liver lesions (Supplementary Figure S1). In contrast, the genes in the CIBERSORTx LM22 are only associated with one type of liver disease.

### 4.2 Concordance between the predicted cell composition and the true proportions

#### 4.2.1 Pure‐cell samples

HCCImm was then assessed by gene expression profile analysis against the known proportions of immune cells. First, each of the 196 microarray samples was tested using the leave‐one‐out strategy as mentioned in the Materials and Methods. Since each microarray sample was derived from only a single type of immune cell, each of these microarray samples was regarded as a pure‐cell sample, and the true cell composition for each of the pure‐cell samples was 100% for its specific type of cell.

**FIGURE 3:**
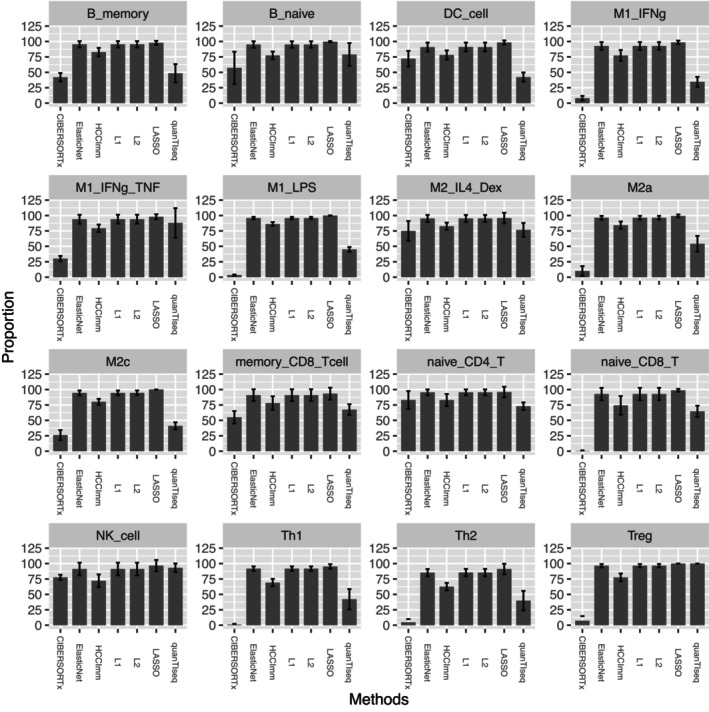
The bar plots for the benchmark using pure‐cell samples. Each graph corresponds to the predicted fraction of the specific cell type of one of the 196 pure‐cell samples. The performance of HCCImm, L1‐ and L2‐regularized linear regression, LASSO, quanTIseq, and CIBERSORTx are shown.

In this benchmark test, the prediction made by HCCImm was able to identify the composition of the major cell type in the pure‐cell samples. The predictions made by HCCImm were consistent with the original data for the seventeen types of cells. HCCImm, as well as the regularized regression approaches using exactly the same refGES built in this study, consistently predicted the dominant cell type with a median fraction of ~80% and short error bars, with the predicted fractions for other cell types being generally lower than 10% (Figure 3). In contrast, the median values for the cell fractions predicted by the two most widely used tools, namely, quanTIseq and CIBERSORTx, were lower than 60%, and there were larger variances.

#### 4.2.2 Memory CD8 ^+^ T samples

To evaluate whether our refGES is suitable for performing cell deconvolution analysis against the GEPs containing heterogeneous subsets of memory CD8^+^ T cells, an independent test was performed by using two additional NCBI GEO datasets that were not used to build our refGES. These two datasets consist of a series of GEPs, where each corresponds to a distinct subset of memory CD8^+^ T cells (NT‐MCD8T, see Materials and Methods, 3.1). Therefore, this assessment can also be conceived as an extended test using GEPs consisting of pure‐cell samples.

First, by carrying out correlation analysis, we noticed that the GEPs of these NT‐MCD8T data were more similar to those of the memory CD8^+^ T cells (MCD8T) used to build our refGES than to those of the naïve CD8^+^ T cells (Supplementary Figure S5). Next, we predicted the cell compositions of these NT‐MCD8T data using a linear regression approach, LASSO. We found that LASSO regression consistently predicted the dominant cell type as MCD8T, with a high median fraction of ~74% and a narrow interquartile range (IQR), and the sum of the predicted fractions for other cell types was generally lower than ~34% (Supplementary Figure S6). The LASSO regression result suggests that even though some subsets of memory CD8^+^ T cells may not perfectly match the features of MCD8T cells used in the refGES, our refGES is suitable for analyzing GEPs that contain such memory CD8^+^ T‐cell subsets.

We used two sets of DEGs for further analysis. One DEG set was prepared by comparing the GEPs of memory CD8^+^ T cells and naïve CD8^+^ T cells. The other DEG set was prepared by comparing the GEPs of PD1‐high CD8^+^ T cells (for the GEO accession, see Table S2) and naïve CD8^+^ T cells. The significantly enriched pathways in both DEG sets are involved in the regulation of exhausted CD8^+^ T cells (Supplementary Figure S7), including calcium signaling,^57^ mitochondrial dysfunction,^58^ the sirtuin signaling pathway^59^ and glucocorticoid receptor signaling.^60^

#### 4.2.3 PBMC microarray and RNA‐seq samples

Unlike the previous evaluation of our method, where only pure‐cell samples were used, the next set of benchmark testing was based on real human samples consisting of multiple cell types, namely, human PBMCs with linked flow cytometry results. The purpose of this test was to evaluate whether HCCImm still performs well when the samples are derived from real human tissues. Importantly, however, in these samples, the cell types that had been quantified were not completely consistent with those that could be predicted by HCCImm and by the other CCD tools. For example, in the GSE107990 dataset, only the fraction of the precursor of macrophages, namely, monocytes, had been determined, but not the different polarization forms of macrophages. Another example is that only CD4^+^ T cells were experimentally determined for this PBMC microarray dataset, whereas HCCImm is able to predict the fractions of five subtypes of CD4^+^ T cells, including naïve CD4^+^ T, Treg, Th1, Th2, and memory CD4^+^ T cells. To compare the predicted fractions with the original differential cell type data during this benchmark, the fractions predicted by HCCImm for various polarization forms of macrophage M1 and M2 cells were summed to give an estimate of the total monocyte fraction in the samples. The full table mapping the predicted cell types against the experimental cell type dataset is provided in Table S4(A).

**FIGURE 4:**
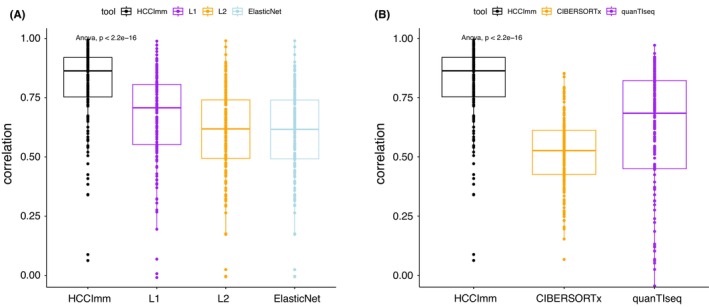
Boxplots of the correlation coefficients of the predictions against the human PBMC microarray sample dataset GSE107990. Elastic, L1, and L2 correspond to the linear regression methods subject to elastic net, L1‐regularization, and L2‐regularization, respectively. (A) Boxplots of the Pearson's correlation coefficients for HCCImm, L1‐, L2‐, and elastic net‐regularized methods. (B) Boxplots of Pearson's correlation coefficients for HCCImm, CIBERSORTx, and quanTIseq.

The results show that HCCImm was superior to the other regularized linear regression approaches tested in this study, as well as publicly available tools such as quanTIseq and CIBERSORTx. Specifically, our approach has a significantly higher median value for the correlation coefficients (~0.86) between the predictions and the experimental PBMC microarray datasets (Figure 4).

**FIGURE 5:**
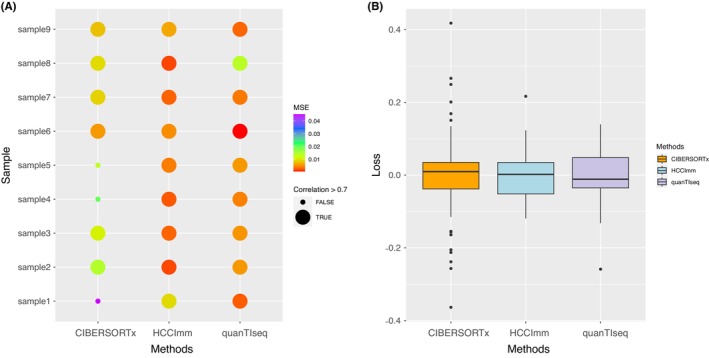
(A) Corrplot of Pearson's correlation coefficients and (B) boxplots of the prediction errors using the experimental cell fractions from the human PBMC RNA‐seq dataset.

However, when the nine RNA‐seq samples from human PBMCs with known cell fractions were used for benchmarking, it could be seen that the predictions made by quanTIseq, a method originally designed to analyze RNA‐seq data, had a high correlation with the experimental cell fractions. In this benchmark test, the performance of HCCImm was better than that of quanTIseq, with the median values of the correlation coefficients being ~0.88 and ~ 0.85, respectively, whereas higher variances in the correlation coefficients can be seen for the predictions made by CIBERSORTx. The predictions made by HCCImm have lower mean square errors (MSEs) than those of the other two methods (Figure 5A). The median error of the predictions made by HCCImm was closer to zero (Figure 5B). The assessment suggests that both quanTIseq and our ε‐SVR‐based method, HCCImm, outperform CIBERSORTx when real human RNA‐seq samples are used. When using the expression signals derived from multiple cell types, the quanTIseq approach could quantify only ten types of immune cells. Although the refGES used in this study was originally built to utilize microarray samples, this benchmark test supports that HCCImm can also be applied to analyze RNA‐seq samples.

### 4.3 Simulated bulk tissues with different fractions of HCC cells

Next, an association analysis between the immune cell compositions and the survival of HCC patients, as well as other cancer features, was performed. This part of the study was designed to take advantage of the gene expression data of the TCGA‐LIHC dataset along with the available clinical data, including survival time. A benchmark test to reveal the performance of HCCImm when applied to the bulk expression profiles of HCC samples was developed. However, bulk GEPs are seldom accompanied by experimentally determined cell fractions. Therefore, we simulated the bulk expression profiles of the HCC samples to assess our CCD method.

**FIGURE 6:**
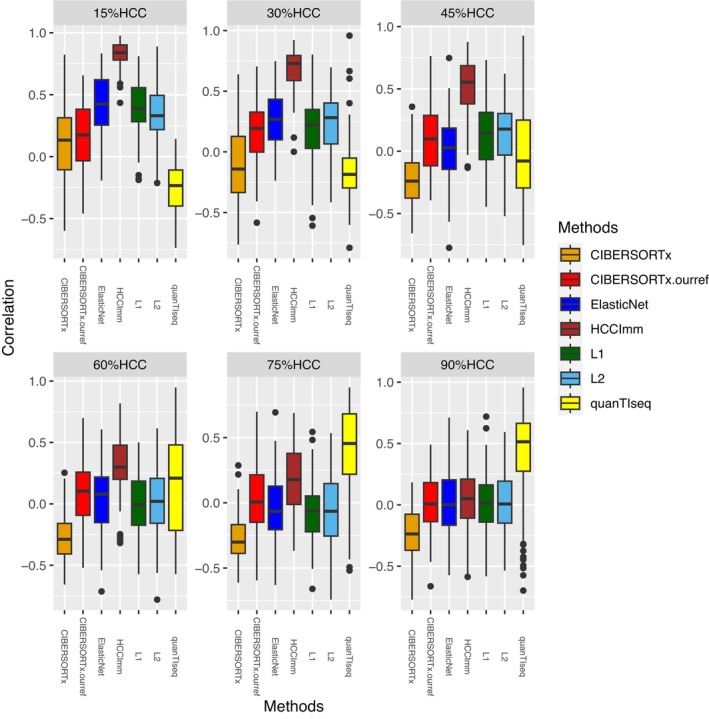
Boxplots of Pearson's correlation coefficients of the stimulated dataset predictions compared to the original data. The plots were generated using the simulated bulk HCC samples for each benchmark based on the gene expression signals with a fixed fraction of HCC cells, namely, 15%, 30%, 45%, 60%, 75%, and 90%. Each data point in each boxplot represents a simulated HCC sample.

**FIGURE 7:**
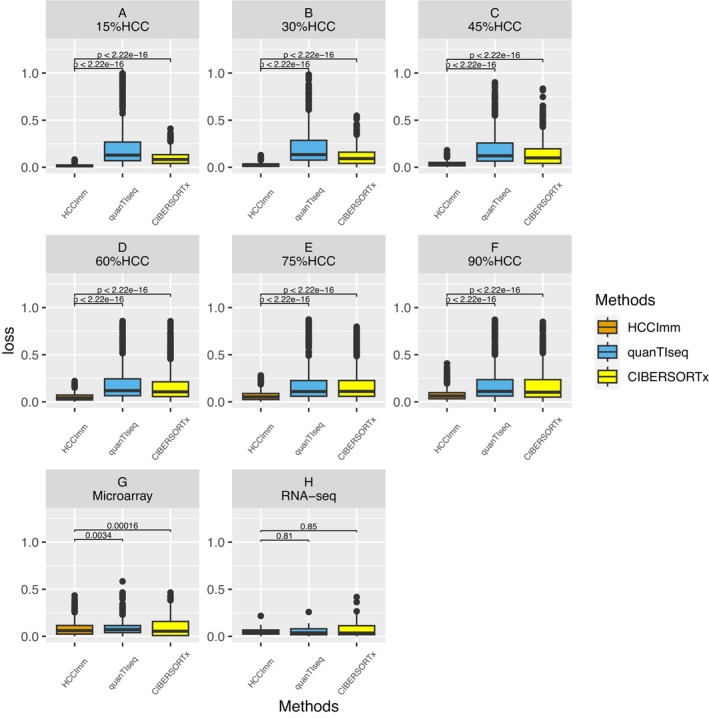
Boxplots of the predicted cell fraction absolute errors (AEs). A–F: The boxplots correspond to each benchmark based on the gene expression signals with a fixed HCC cell fraction, namely, 15%, 30%, 45%, 60%, 75%, and 90%, in the simulated bulk tissues. G,H: The boxplots correspond to the benchmark tests using the microarray and RNA‐seq samples of PBMCs, respectively.

Since the dominant cell type in the TME is most likely tumor cells, they should make up a high proportion of the bulk GEP. Therefore, we created synthetic datasets that contained a fixed percentage (*x*%) of gene expression signals derived from HCC cells, and the remaining fraction (100% ‐ *x*%) was composed of signals from the sixteen types of immune cells that were randomly sampled and weighted. The results revealed that HCCImm, our ε‐SVR‐based method, was quite robust, showing consistently high correlations between the predicted cell fractions and predefined fractions of tumor cells (Figure 6). Although the predictions made by quanTIseq had higher correlations with original data, when the fractions of HCC cells in the simulated datasets were higher than 75% (Figure 6), the predictions made by HCCImm had significantly smaller AEs than those of the predictions made by quanTIseq and CIBERSORTx (Figure 7A–F).

Furthermore, to evaluate the effect of refGES on the cell deconvolution model, we utilized our refGES instead of the default LM22 matrix to carry out CIBERSORTx analysis, and this test was labeled CIBERSORTx.ourref. Based on the correlation between the CIBERSORTx.ourref result and the original data of the simulated datasets, we determined that its performance was better than that of CIBERSORTx with default LM22, albeit still not as robust as HCCImm.

However, for the previous benchmark using pure‐type samples, the performances of *L1*‐regularized, *L2*‐regularized, and elastic net‐regularized linear regressions were slightly better than that of HCCImm (Figure 3). This benchmark using simulated HCC samples supports the hypothesis that our ε‐SVR‐based method, HCCImm, together with the new refGES, is likely to be more suitable for analyzing HCC bulk tissue samples that contain multiple cell types.

### 4.4 Association of immune cell abundance with HCC patient survival and TMB

The ultimate goal of this study was to explore whether there is an association between immune cell abundance and the clinical features of HCC patients, such as survival time and TMB. Among the TCGA‐LIHC patients, 369 patients had complete survival information, and these patients were extracted for cell fraction prediction (Figure 1C,D). The raw counts of the RNA‐seq samples of these patients were converted to TPM values and transformed as mentioned in the “Methods” section.

**FIGURE 8:**
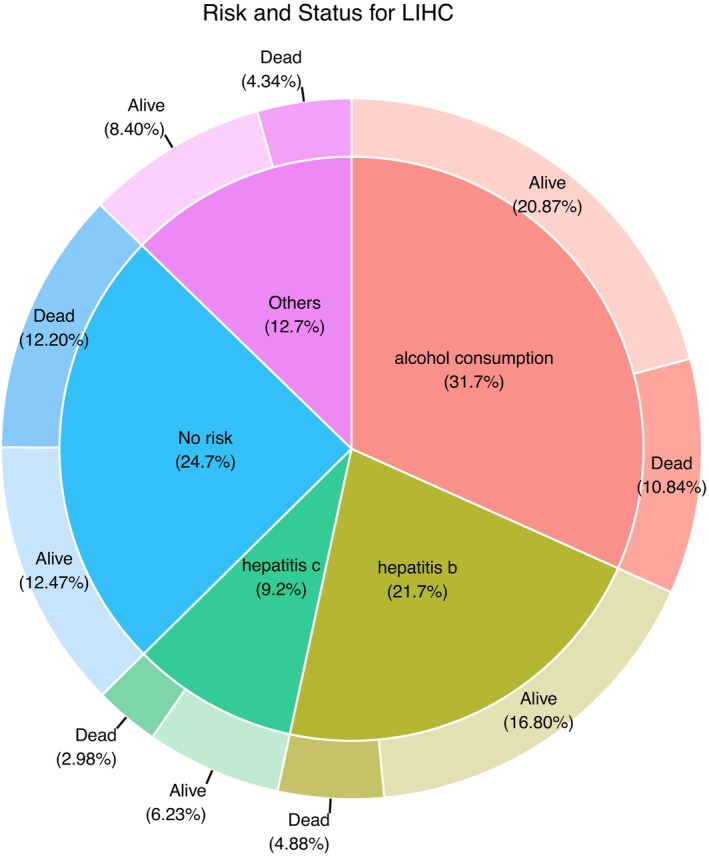
TCGA‐LIHC patients were categorized into four risk groups.

**FIGURE 9:**
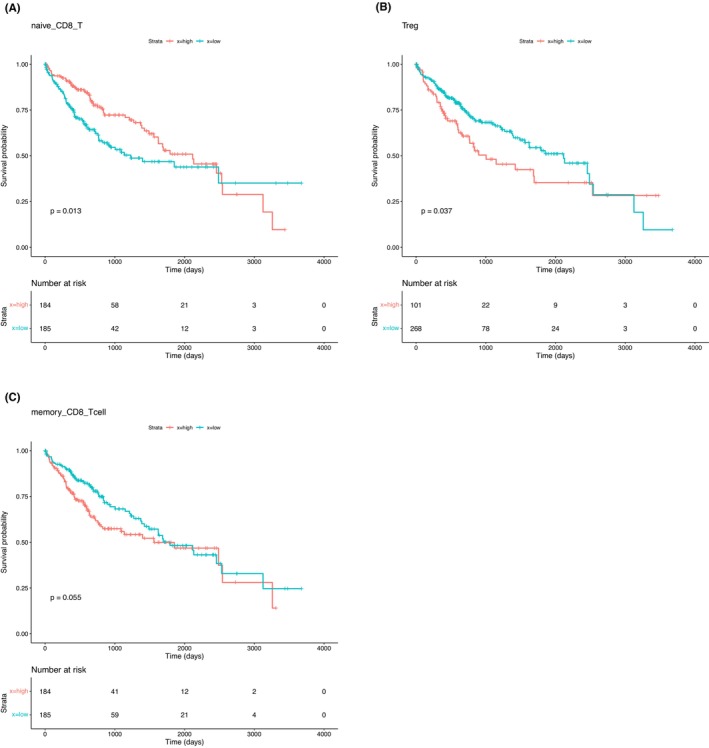
Kaplan–Meier survival curves of HCC patients were created by dividing patients into high and low groups corresponding to high and low abundances of naïve CD8^+^ T cells, Treg cells, and memory CD8^+^ T cells, A, B, and C, respectively. The red lines indicate the high group, while the green lines indicate the low group.

The strategy used for the association analysis between the immune cell fractions and patient survival is as follows. First, the cell fractions of the 369 TCGA‐LIHC RNA‐seq samples were predicted. The patients were then categorized into four groups based on their risk histories (Figure 8). The association of the cell fractions with the OS of the HCC patients was then assessed by the log‐rank test and univariate Cox regression analyses (Figure 1D). To consider the potential bias when performing cross‐sample comparisons of the abundance of each immune cell type, we decided that the predicted HCC cell fractions should be removed and the remaining fractions of the sixteen types of immune cells in each sample were then normalized to 100% (Figure 1C). We argue that the potential variations in the resection procedure during tumor sample acquisition may have affected the RNA‐seq results, such that the proportion of gene expression signals derived from HCC cells across different samples. For the respective association analysis between immune cell abundance and patient prognosis, the Kaplan–Meier method was used to create survival curves. For each immune cell type, the 369 HCC patients were divided into high and low groups. The high group represented the patients with a predicted fraction of one immune cell type higher than the median value, and vice versa. By using this approach, it was found that the abundance of three immune cell types was significantly associated with HCC patient OS (Figure 9). HCC patients with higher fractions of memory CD8^+^ T cells and Treg cells had a worse prognosis (log‐rank test *p‐*values of 0.055 and 0.037, respectively). In contrast, HCC patients with a higher fraction of naïve CD8^+^ T cells were found to have a significantly better prognosis (log‐rank test *p‐*value: 0.013). In addition, for subgroups of HCC patients carrying risk factors, including HCV and alcohol consumption, a higher fraction of Tregs and a higher fraction of memory CD8^+^ T cells were associated with a worse prognosis (Supplementary Figure S8).

The Kaplan–Meier analysis indicated that the higher abundance group of naïve CD8^+^ T cells was a significant factor for the survival of HCC patients (Figure 9). Two subtypes of CD8^+^ T cells are associated with HCC patient prognosis. Patients were divided into four groups based on their proportions of naïve and memory CD8^+^ T cells: higher proportions of naïve CD8^+^ T cells and higher proportions of memory CD8^+^ T cells (naïve_h&mem_h), higher proportions of naïve CD8^+^ T cells and lower proportions of memory CD8^+^ T cells (naïve_h&mem_l), lower proportions of naïve CD8^+^ T cells and higher proportions of memory CD8^+^ T cells (naïve_l&mem_h), and lower proportions of naïve CD8^+^ T cells and lower proportions of memory CD8^+^ T cells (naïve_l&mem_l). The Kaplan–Meier plot analysis revealed the association between these CD8^+^ T‐cell subtypes and patient prognosis. The results showed that patients with a higher proportion of naïve CD8^+^ T cells had a better prognosis, regardless of the memory CD8^+^ T cell proportion. This finding suggests that the proportion of naïve CD8^+^ T cells could serve as a valuable prognostic marker for HCC patients (Supplementary Figure S9).

**FIGURE 10:**
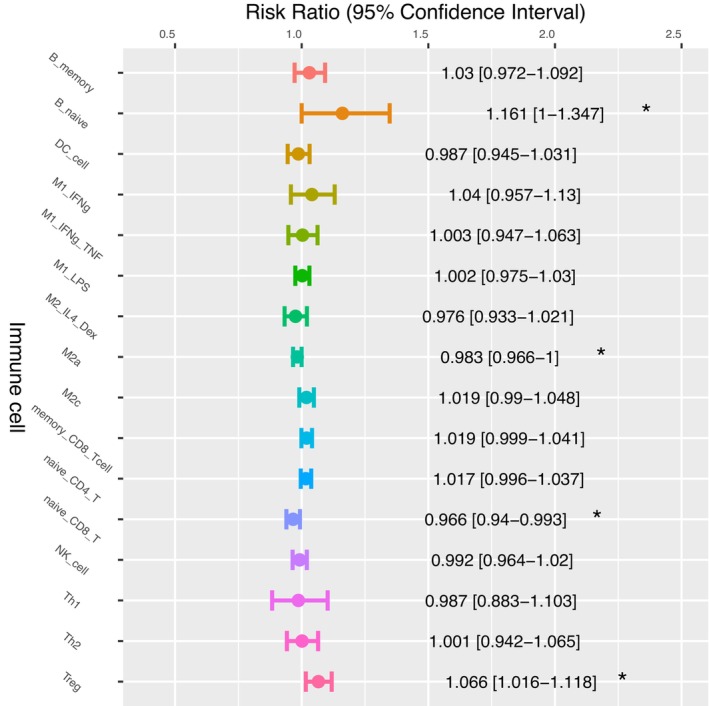
Cox regression analysis between immune cell abundance and the OS of 369 HCC patients using the proportions of sixteen immune cell types as the independent variable.

To further investigate which immune cell types might be critical to HCC patient survival, univariate Cox regression analysis (Cox, 1972) was performed to assess whether there was a correlation between immune cell fractions and HCC patient OS. The hazard ratios (HRs) and 95% confidence intervals (CIs) of the sixteen immune cell types are shown in Figure 10. These results revealed that two types of immune cells, M2a cells, and naïve CD8^+^ T cells, seem to have a positive impact on HCC patient OS. In contrast, memory CD8^+^ T cells seem to have a negative impact on HCC patient OS (*p‐*value = 0.07). Furthermore, higher abundances of Treg cells and naïve B cells were found to be significantly associated with a worse OS (Treg: HR = 1.07, 95% CI = 1.02–1.12, *p‐*value = 0.01) (B_naïve: HR = 1.16, 95% CI = 1.00–1.35, *p‐*value = 0.05).

Moreover, progression‐free survival (PFS), in addition to OS, can also provide valuable information, which shows how long a patient's cancer remains stable or does not worsen after the initial diagnosis.^61^ The results revealed that in the TCGA‐LIHC patients, the proportions of naïve CD8^+^ T cells and Tregs were significantly associated with PFS. Naïve CD8^+^ T cells seem to have a positive impact on HCC patient PFS, whereas Treg cells are likely to have a negative impact on HCC patient PFS, suggesting that these immune cell subsets may play a role in cancer progression and the response to treatment (Supplementary Figure S10).

**FIGURE 11:**
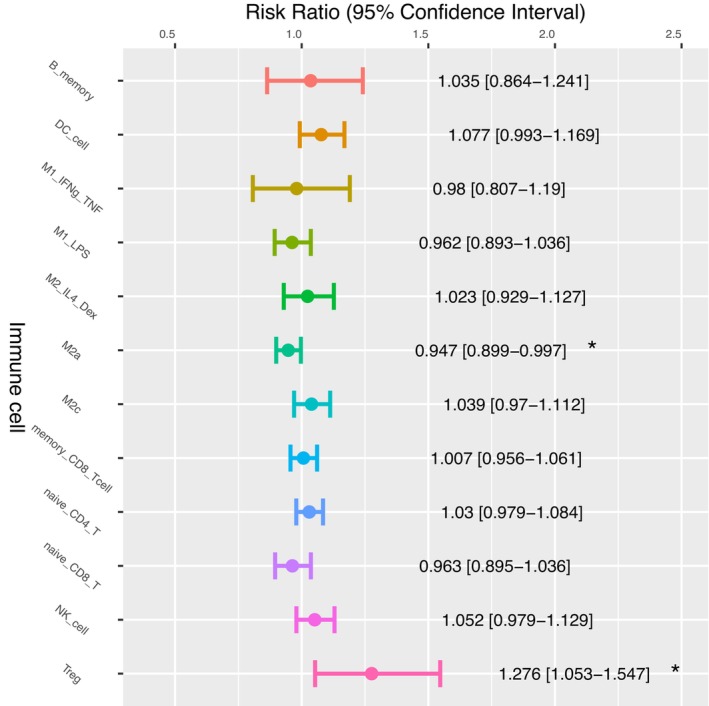
Cox regression analysis of immune cell abundance in HBV‐HCC patients using the fraction of 16 immune cell types as the independent variable.

We further categorized the HCC patients into distinct risk groups based on factors such as chronic hepatitis and alcohol consumption. Each risk group is likely to represent a distinct type of underlying condition related to chronic inflammation. For example, hepatitis B virus (HBV) carriers often suffer from chronic inflammation of the liver due to recurrent reactivation of their HBV infection,^62^ and HBV‐specific CD8^+^ T cells then infiltrate into their liver and acquire a specific phenotype.^63^ Moreover, alcohol consumption might lead to a proinflammatory effect involving Kupffer cells, which has been shown to reduce the recruitment of CD8^+^ T cells.^64^ A number of associations between immune cell abundance and the various risk groups have been identified. For example, the HR of Treg cells in the OS of HBV‐HCC patients is 1.28, which is higher than that of all other HCC patient groups. Interestingly, unlike the survival of HBV‐HCC patients, the abundance of Treg cells does not appear to be significantly associated with the survival of HCC patients in the other risk groups (Figure 11). We noticed that in addition to the HBV group, the survival of the hepatitis C virus (HCV) group might be associated with the abundance of Tregs. A Cox hazard analysis for PFS in the HCV‐HCC group showed a HR = 1.12 and *p*‐value <0.05 for Treg abundance (Supplementary Figure S10). We reviewed the literature and noticed that this association between Treg cell abundance and viral risk group survival might reflect the immunosuppressive role of Treg cells in the progression of chronic viral hepatitis, which might not occur in non‐viral HCC risk groups. In hepatitis viral infections, Treg cells play a protective role in the host by suppressing immune‐mediated mechanisms of liver damage.^65^ Another study also provided evidence that HBV infection‐related factors could lead to the expansion of Tregs and enhance their suppressor function, which in turn could dampen the antitumor immune response to HCC tumor antigens and hinder the immune surveillance of HCC tumors.^66^

**FIGURE 12:**
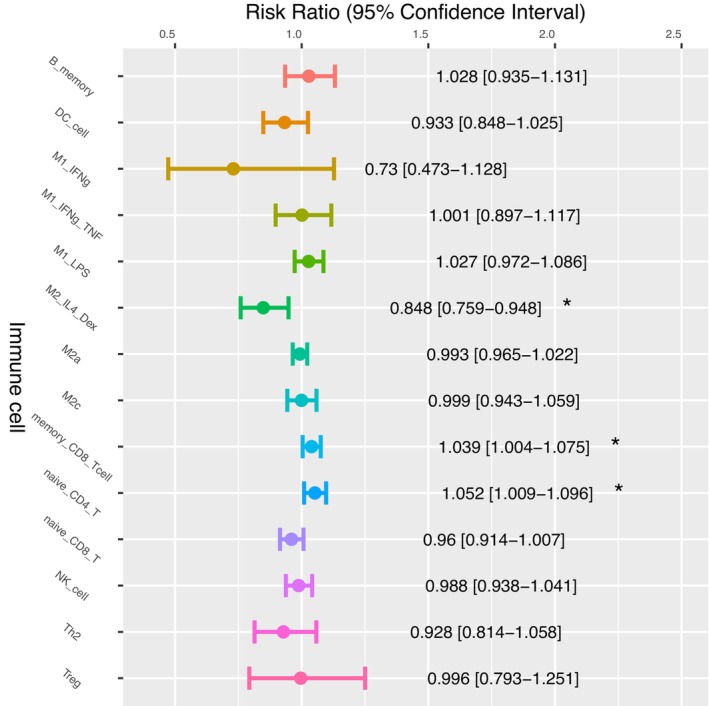
Cox regression analysis of the immune cell abundance of alcohol‐HCC patients using the fraction of 16 immune cell types as the independent variable. Note: *p < 0.05.

Another association was found between the M2 polarization subtypes, M2a and M2_IL4_DEX, and the OS of HCC patients with HBV and alcohol consumption risk factors. The HBV‐HCC patients and the alcohol‐HCC patients who had higher fractions of M2a (Figure 11) and M2_IL4_DEX (Figure 12), respectively, were found to have HRs significantly lower than 1.0. In contrast, the immune cell types associated with increased HRs among alcohol‐HCC patients are different from those associated with increased HRs among HBV‐HCC patients. Alcohol‐HCC patients with a higher abundance of memory CD8^+^ T cells and naïve CD4^+^ T cells have a worse prognosis (Figure 12). However, HCV‐HCC and HCC patients without other risk factors did not have a significant hazed ratio (Supplementary Figures S11 and S12).

### 4.5 Association between immune cell fraction and TMB

Cancer immunotherapy has changed the treatment landscape for cancer patients. However, in terms of the treatment of HCC, only a small subset of patients respond well to immunotherapy.^67^ Therefore, a better understanding of the interaction between HCC cells and immune cells in the TME should provide insights into possible prognostic and predictive biomarkers that can then be used to select patients who might benefit from ICI‐based treatment.^67^ A high TMB has been proposed as a biomarker to predict the response to immune checkpoint blockade (ICB) based on the hypothesis that increased tumor mutations lead to the production of more antigenic peptides, which enhances immunogenicity.^68^ In contrast, available evidence suggests that an association between TMB and immune signatures is dependent on the cancer type, and a high TMB has been associated with a poor prognosis among TCGA‐LIHC patients (Supplementary Figure S13).^6^ Therefore, we investigated whether the TMB level of HCC patients was correlated with the levels of certain immune cell types that might be associated with immunosuppression in the HCC TME.

**FIGURE 13:**
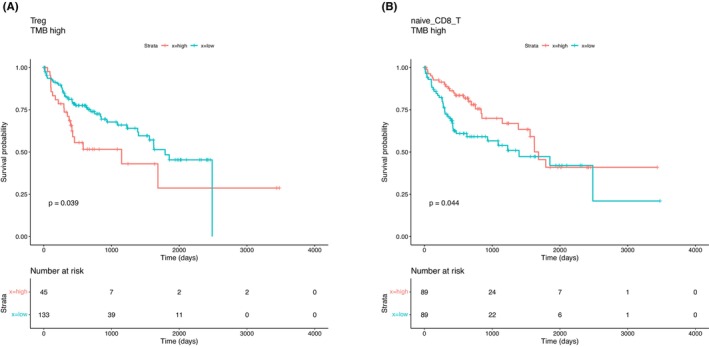
Kaplan–Meier survival curves of HCC patients with high TMB were obtained by dividing the patients into two groups based on their level of (A) Treg cells and (B) naïve CD8^+^ T cells.

We used HCCImm to investigate the association between TMB and the abundance of leukocytes in TCGA‐LIHC samples. The TCGA mutation calls were acquired from the MC3 calls in the Pancancer Atlas (https://gdc.cancer.gov/about‐data/publications/pancanatlas).^54^ We noticed that when the LIHC samples had a high TMB (TMB‐H), there were significantly lower fractions of the M2 cell subtypes (*t*‐test, *p‐*value = 0.017), and this was linked to a significantly higher sum for all of the fractions of the other leukocytes, specifically excluding macrophages (*t*‐test, *p‐*value = 0.001). An association of TMB‐H with M2 cell levels and other tumor‐infiltrating lymphocyte (TIL) cell levels was also observed in the HBV‐HCC patient subgroup. In the TMB‐H group, the subset of patients with a higher fraction of Treg cells had a worse prognosis (log‐rank test *p‐*value: 0.039) (Figure 13A), whereas the subset of patients with a higher fraction of naïve CD8^+^ T cells had a significantly better prognosis (log‐rank test *p‐*value: 0.044) (Figure 13B).

Moreover, to investigate the potential impact of Treg abundance on the relationship between HCC patient OS and their TMB levels, we generated waterfall plots separately for high‐ and low‐Treg groups (Supplementary Figure S14). The y‐axis of the plots represents the estimated survival scale, which was calculated using a min‐max scaling method based on patients' survival time. These plots revealed that, within the first 2 years after diagnosis, HCC patients with high TMB and Treg‐high status experienced a significantly shorter overall survival time (survival scale *y* < 0.2, Fisher's exact test, *p*‐value of 0.017, Supplementary Figure S14A). In contrast, in Treg‐low status, the result shows that the TMB levels were not associated with survival (Supplementary Figure S14B). This finding provides further support for the notion that Treg abundance may play a role in the progression of HCC in patients with high TMB levels.

### 4.6 Immune cell levels and anticancer drug response among TCGA‐LIHC patients

We explored the association between immune cell abundance and patient response to treatment. Although data on the responses to ICB therapy are not available for TCGA‐LIHC patients, a few records about the responses to cancer therapy have been documented in TCGA clinical data. Previously, Ding et al. curated drug treatment information of TCGA patients (Supplementary Figure S15), wherein 180 clinical response records related to four chemotherapeutic drugs were obtained to determine the molecular features that can predict outcomes.^54^ Here, we took advantage of these records to assess the predictive utility of immune cell levels with respect to clinical drug responses among TCGA‐LIHC patients. Interestingly, all four of the LIHC patients that showed complete or partial responses to treatment had higher levels of DCs and naïve CD8^+^ T cells (Fisher's exact test, *p‐*value = 0.057) (Table 2A). When we focused only on the HCC patient subgroup that had a high level of memory CD8^+^ T cells or Tregs, a high abundance of naïve CD8^+^ T cells was significantly associated with a greater treatment response (Fisher's exact test, *p‐*value = 0.042) (Table 2B).

**TABLE 2:** The 2 × 2 tables for TCGA‐LIHC patients with drug response records have high and low levels of naïve CD8^+^ T cells. (A) All LIHC patients. (B) The HCC patient subgroup with a high level of memory CD8^+^ T cells or Tregs.

A		
	Responder	No treatment records
High naïve CD8^+^ T	4	169
Low naïve CD8^+^ T	0	180

B		
	Responder	No treatment records
High naïve CD8^+^ T	3	70
Low naïve CD8^+^ T	0	135

## 5 DISCUSSION

### 5.1 Bulk RNA‐seq and differential polarizations

Our ε‐SVR‐based method, HCCImm, was designed to use the bulk GEPs of HCC samples to predict the abundance of different major immune cell types such as B cells, CD8^+^ T cells, CD4^+^ T cells, and macrophages. The benchmark testing revealed that HCCImm had at least a comparable performance to other immune cell quantification methods when used to analyze bulk RNA‐seq samples. Furthermore, HCCImm was also able to make predictions for more cell types than earlier methods, such as quanTIseq, which was specifically designed to analyze RNA‐seq data. In addition, when analyzing the TCGA‐LIHC dataset, for >95% (355/369) of the cases, our predictions for cell composition passed the Monte Carlo test (*p‐*value <0.05). Therefore, HCCImm was able to predict the fractions of immune cell subtypes, which were then correlated with HCC patient OS.

### 5.2 Correlation between OS, treatment response, and immune cell composition

One important issue concerns the association between TAMs and HCC patient OS. TAMs with the M2 phenotype have been proposed to be key players in cancer‐related inflammation and are involved in promoting the survival, growth, and metastasis of cancer cells, as well as the suppression of adaptive immunity.^69^ However, in previous computational studies investigating immune cell composition, there have been no consistent findings with respect to an association between the level of macrophage polarization and HCC patient OS. For example, Manoharan et al. reported that a high level of monocytes, but not macrophages, was negatively associated with TCGA‐LIHC patient OS.^70^ Foerster et al. stated that the absence of macrophages and Th2 cells were positively correlated with TCGA‐LIHC patient OS. Peng et al. found that TCGA‐LIHC patients in the poor prognosis group had higher levels of M0 and M2 cells.^33^

Using HCCImm, we have been able to predict the cell fractions in the TCGA‐LIHC samples; this analysis included multiple polarized forms of macrophages, not just the binary M1 and M2 cell categories. After the analysis was carried out, we found no significant association with patient OS. Hourani et al.^71^ reported that the subtypes of M2 macrophages that are associated with poor cancer prognosis differ across various cancer types. In liver cancer, M2 cells have been commonly associated with poor prognosis. However, it remains unclear how the subtypes of M2 cells specifically impact different types of liver cancer. When we further investigated the subgroups of HCC patients with distinct risk histories, the HBV‐HCC patients and alcohol‐HCC patients with higher levels of M2a (Figure 11) and M2_IL4_DEX (Figure 12), respectively, were found to have HRs significantly lower than 1.0. This result suggests that not all subsets of M2 cells are associated with a poor HCC patient prognosis.

This result seems to contradict the common viewpoint that TAMs with an M2 phenotype are associated with a poor prognosis among HCC patients. Perhaps because we have been unable to find a negative association of any macrophage subtype with HCC patient OS, the results might reflect the possibility that the selected macrophage subtypes in this study do not correspond well to the survival‐associated subsets of macrophages. Since TAMs are composed of heterogeneous subsets of functionally distinct macrophages, perhaps only a subset of TAMs might have an important association with patient survival, which is supported by recently published research using scRNA‐seq to investigate immune cells in the HCC TME.^72^ Nonetheless, our results also indicate that some subsets of macrophages may improve the prognosis of several HCC patient subgroups. Notably, several meta‐analyses have also suggested that TAMs are not necessarily associated with poor cancer patient prognosis.^73^, ^74^, ^75^

CD8^+^ T cells are another type of immune cell that is critical for tumor control and is known to be associated with a favorable HCC prognosis.^72^, ^76^ Our analyses using the log‐rank test and Cox univariate regression revealed that HCC patients with a higher fraction of naïve CD8^+^ T cells had a favorable prognosis. In contrast, the abundance of another CD8^+^ T‐cell subset, memory CD8^+^ T cells, seems to be associated with a HR significantly larger than 1.0 (Figure 10). This result suggests that in the HCC TME, there is a positive association between memory CD8^+^ T cells and patient OS, which is similar to that found for Treg immunosuppressive cells (Figure 10). At first glance, this finding also appears to contradict our current knowledge related to tumor‐infiltrating cytotoxic CD8^+^ T cells, which have been shown to suppress the growth of tumor cells and therefore should be beneficial for patient OS. Nevertheless, CD8^+^ T cells are known to be able to differentiate into exhausted T cells within the TME of various cancer types, including HCC. Furthermore, memory CD8^+^ T cells in the HCC TME seem to present with a T‐cell exhaustion phenotype.^19^ Our results showing the negative association between memory CD8^+^ T cells and HCC patient OS might reflect the suppression of CD8^+^ T cell cytotoxicity via the activation of the immune checkpoint pathway by PD‐L1^+^ cells (Supplementary Figure S16). Interestingly, based on the predictions made by HCCImm, we also found that a high level of naïve CD8^+^ T cells in the HCC TME seems to be associated with patient response to cancer treatment (Table 2), although the available data are fairly limited. Overall, in the HCC TME, our results support the hypothesis that naïve CD8^+^ T cells have a positive impact on patient survival and drug response.

### 5.3 Correlation of immune cell levels with TMB in TCGA‐LIHC patients

By applying CIBERSORT to quantify 22 immune cell types, McGrail et al. reported that in TCGA‐LIHC samples, the abundance of CD8^+^ T cells was not positively correlated with neoantigens.^68^ A similar result was found in our study; however, we additionally assessed the association between TMB and other immune cell types. When assessing all 369 HCC patients, a significant association of TMB‐H with the abundance of M2 cells and other TIL cells was found. The same association was also found in the subgroup of HBV‐HCC patients, although it was absent when subgroups with different risk histories (i.e., HCV, alcohol, and no history of risk factors) were assessed. The significant association of TMB‐H with TIL cells in TCGA‐LIHC patients has also been previously reported by Wang and Li.^6^

The association between TMB‐H and TIL cells in HBV‐HCC patients may suggest that during the chronic inflammation caused by the HBV, immune cells such as HBV‐specific CD8^+^ T cells directly attack infected hepatocytes and subsequently recruit other components of the immune system, which then leads to immunopathogenesis and further hepatic damage.^63^ Since there is no such finding for HCV‐HCC patients and alcohol‐HCC patients, one interpretation is that distinct histories in terms of risk factors can lead to a range of different carcinogenesis mechanisms. For example, chronic alcohol consumption decreases the number of antitumor CD8^+^ T cells.^77^

Yan et al.^77^ investigated the abundance of antitumor CD8^+^ T cells in alcohol‐HCC patient samples and found that the advanced TME typically contains fewer of these cells than the early TME. In chronic HCC caused by HCV infection, CD8^+^ T cells were found to be exhausted.^78^ Using the HCCImm algorithm to analyze TCGA‐LIHC data, we found a significant difference in the abundance of naïve CD8^+^ T cells between early‐stage (stage I) and advanced‐stage (stage III and IV) alcohol‐related liver cancer. Specifically, our study revealed a lower abundance of naïve CD8^+^ T cells in the advanced stage, suggesting a depletion of this cell population as the disease progresses (Figure S17).

Our results revealed that TMB‐H HCC patients, particularly the subgroup consisting of HBV‐HCC patients, are likely to have significantly higher levels of TILs and significantly lower levels of M2 macrophages. Therefore, HBV‐HCC patients with a high TMB might be a more suitable target group for ICB therapy than the HCC patient groups that have other risk histories, such as hepatitis C and chronic alcohol consumption.

## 6 CONCLUSION

We built a new CCD tool, HCCImm, to estimate the abundance of sixteen immune cell types present in HCC gene expression samples. We have demonstrated its performance by analyzing our results against experimentally known profiles, including microarray and RNA‐seq profiles. This benchmark testing showed that HCCImm is a more robust approach compared to other available CCD methods. By applying this new tool to analyze TCGA‐LIHC samples and conducting various survival analyses, we found that there is a significant association between the abundance of naïve CD8^+^ T cells and memory CD8^+^ T cells and HCC patient prognosis. In addition, we also noticed that patients with a high TMB and a high abundance of naïve CD8^+^ T cells had significantly better prognoses. Moreover, we found that the response of HCC patients to cancer treatment might be positively associated with the abundance of naïve CD8^+^ T cells. Overall, HCCImm enables a robust and comprehensive exploration of immune cell composition in the HCC TME, and our findings are highly consistent with results derived from experimental investigations, which have generally focused on only a limited range of immune cell types.

## AUTHOR CONTRIBUTIONS

**Yen Jung Chiu:** Conceptualization (equal); data curation (equal); formal analysis (equal); investigation (equal); methodology (equal); project administration (equal); resources (equal); software (equal); supervision (equal); validation (equal); visualization (equal); writing – original draft (equal); writing – review and editing (equal). **Chung‐En Ni:** Validation (equal). **Yen‐Hua Huang:** Conceptualization (equal); funding acquisition (equal); investigation (equal); methodology (equal); project administration (equal); supervision (equal); validation (equal); writing – original draft (equal); writing – review and editing (equal).

## FUNDING INFORMATION

This work was supported by grants from the Ministry of Science and Technology (MOST), Taiwan (MOST108‐2320‐B‐010‐041, MOST109‐2221‐E‐010‐017‐MY3), and the Higher Education Sprout Project by the Ministry of Education (MOE), Taiwan (108 AC‐D102).

## CONFLICT OF INTEREST STATEMENT

The authors declare that they have no conflicts of interest.

## ETHICS APPROVAL AND CONSENT TO PARTICIPATE

Not applicable.

## Supporting information


Figure S1–S17.

Table S1.

Table S2.

Table S3.

Table S4A–C.

Table S5.

## Data Availability

Not applicable.
