# Supplementary material for: Deconvolution of bulk gene expression profiles reveals the association between immune cell polarization and the prognosis of hepatocellular carcinoma patients
Source: Cancer Med. 2023 Jun 27;12(14):15736–60. doi: 10.1002/cam4.6197 (PMC10417088; doi:10.1002/cam4.6197)
Supplement: Supplementary file 1 — Figure S1–S17. [file CAM4-12-15736-s006.pdf]

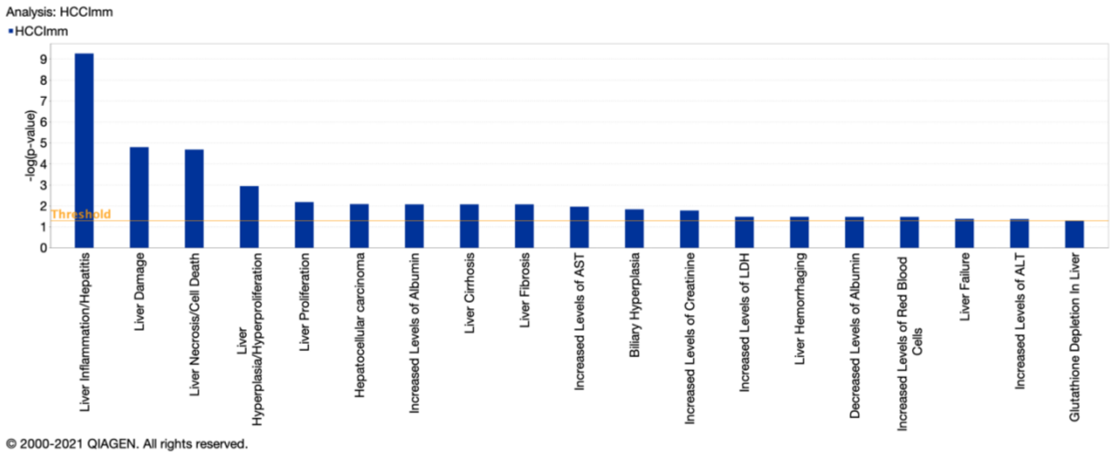

Supplementary Figure 1. Disease and function annotation of reference genes used in the HCCImm analysis. The -logP value was used to evaluate the significance of the network. The threshold was a cutoff p value of 0.05. Next, we used QIAGEN's Ingenuity® Pathway Analysis (IPA®, QIAGEN Redwood City, CA, USA) to identify the diseases and functions associated with these genes.

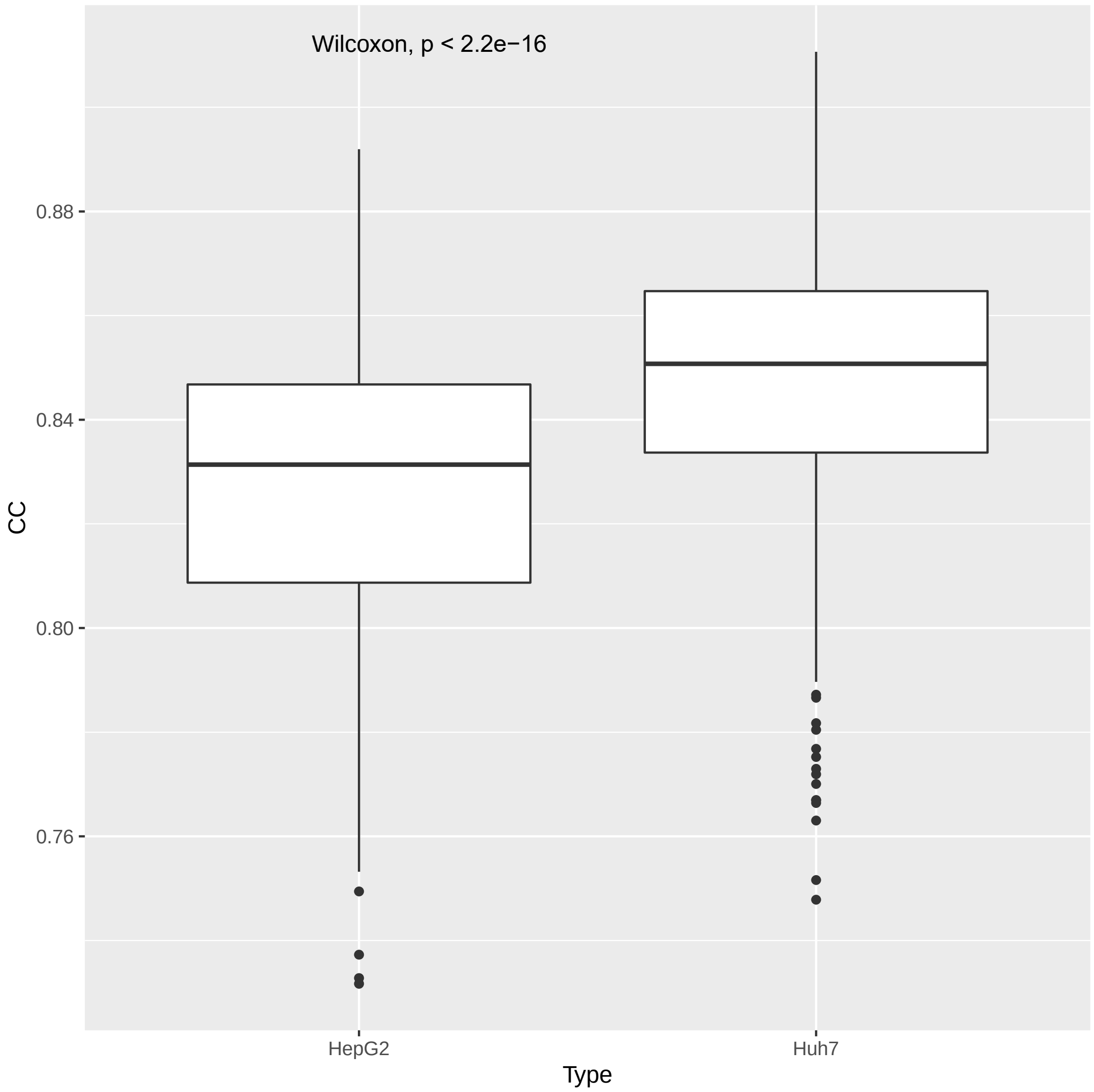

Supplementary Figure 2. Boxplots revealing the correlation of 50 human HCC samples with the HCC HepG2 and Huh7 cell lines. There was a significant difference between the two groups (Wilcoxon signed-rank test, p value < 0.05). This result suggests that human HCC tumors are more similar to Huh7 cells than to HepG2 cells.

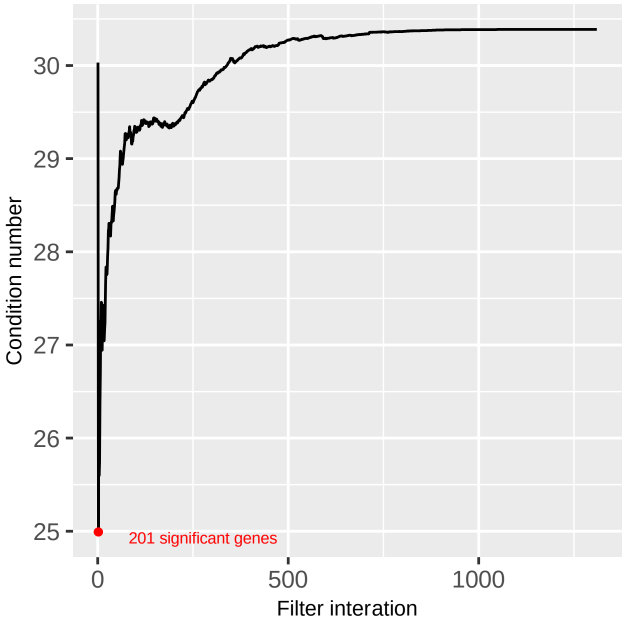

Supplementary Figure 3. Minimization of the condition number to select the top DEGs ranked by p value to build the reference gene expression signature (refGES). The red circle indicates the location of the minimum condition number, where the number of genes is 201.

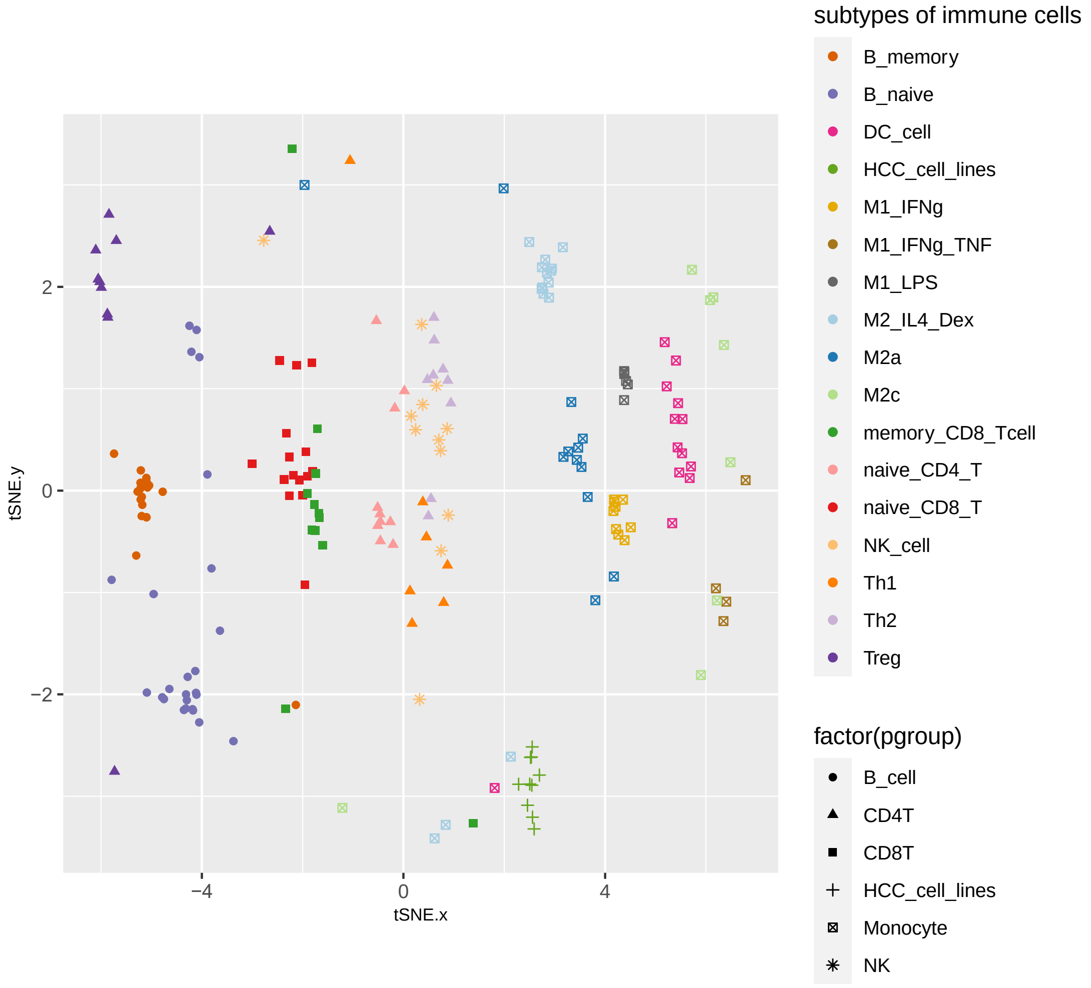

Supplementary Figure 4. t-SNE plot of the 196 microarray samples using all of the signature genes as features. Each data point represents one sample and is color coded according to its cell type.

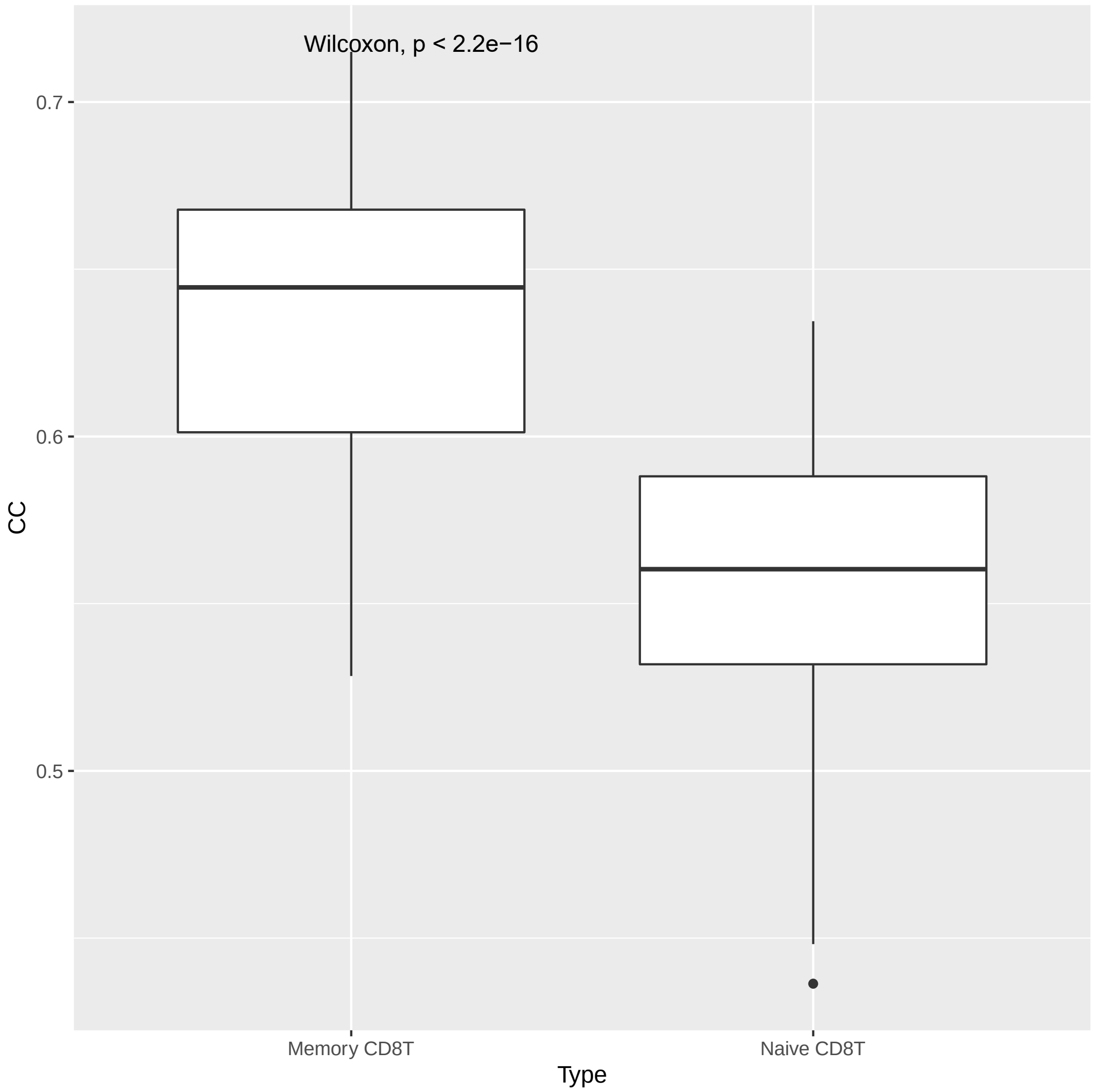

Supplementary Figure 5. Boxplots revealing the correlation of NT-MCD8T samples with the two reference sets of memory CD8⁺ T cells (refMCD8T) and naïve CD8⁺ T cells (refNCD8T), which have been used to build the refGES of HCCImm. Nontypical memory CD8⁺ T cells (NT-MCD8T) were referred to as the memory CD8⁺ T cells of heterogeneous subtypes (see Materials and Methods). Each NT-MCD8T sample carried a distinct gene expression pattern that deviated from the reference MCD8T samples. This diagram shows that there is a significant difference between the distributions of the correlations of NT-MCD8T with refMCD8T and refNCD8T (Wilcoxon signed-rank test, p value < 0.05).

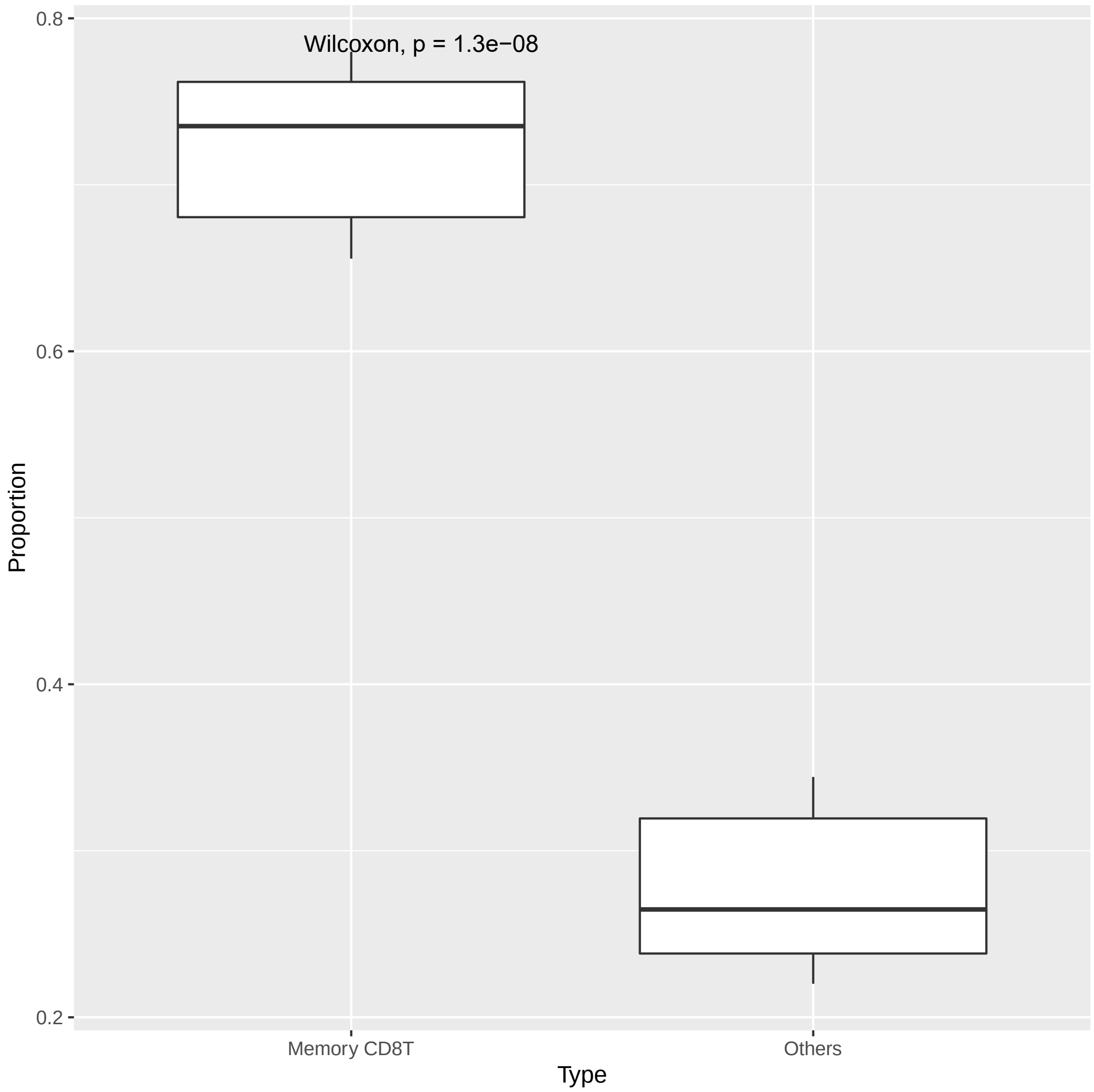

Supplementary Figure 6. Boxplots revealing the distributions of LASSO-predicted cell fractions of "memory CD8$^+$ T cells" (MCD8T) in the NT-MCD8T samples. "Others" represented the sum of the LASSO-predicted fractions of all the other immune cell types. Nontypical memory CD8$^+$ T cells (NT-MCD8T) were referred to as the memory CD8$^+$ T cells of heterogeneous subtypes (see Materials and Methods and the description in Supplementary Figure 5). This diagram shows that there is a significant difference between the LASSO-predicted fractions for MCD8T and for others (Wilcoxon signed-rank test, p value < 0.05).

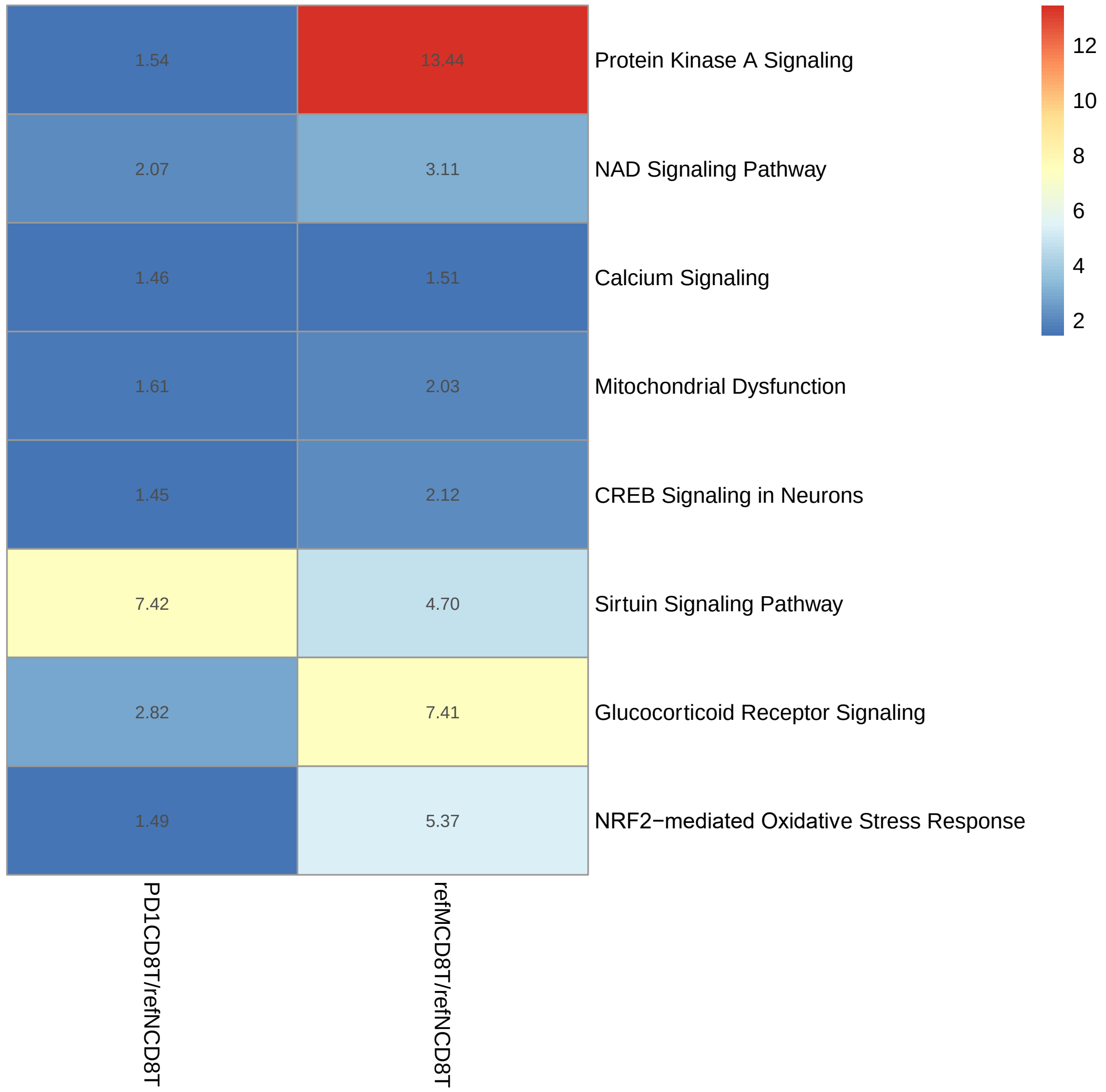

Supplementary Figure 7. Canonical pathway comparison of two gene sets. The -logP value was used to evaluate the significance of the network. The threshold was a cutoff p value of 0.05. Next, we used QIAGEN's Ingenuity® Pathway Analysis (IPA®, QIAGEN Redwood City, CA, USA) to identify the canonical pathways associated with these genes.

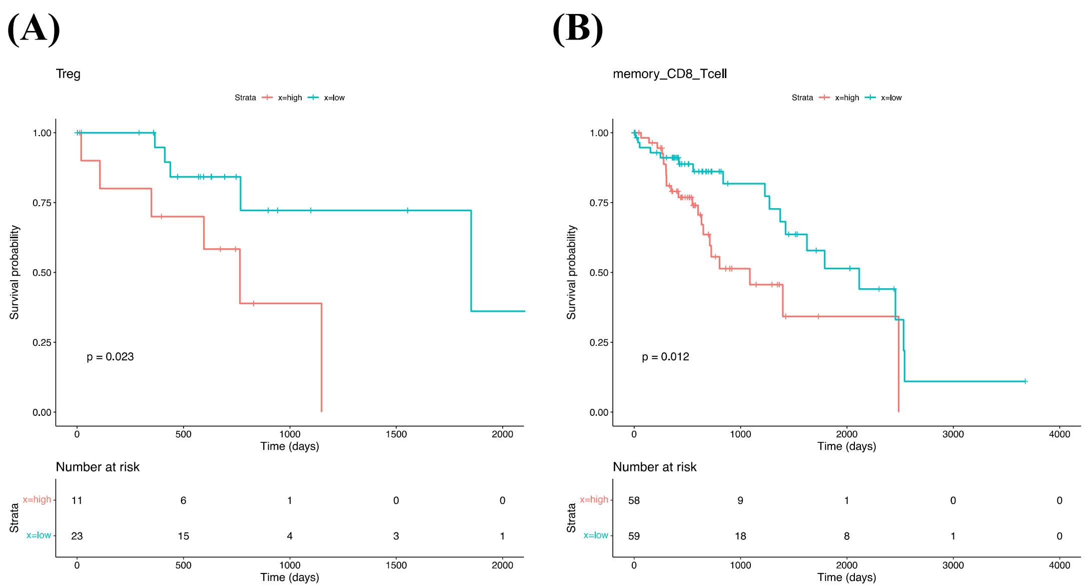

Supplementary Figure 8. Kaplan–Meier survival curves (A) Kaplan–Meier survival curves for TCGA-LIHC HCV-HCC patients with respect to the predicted levels of Treg cells; (B) Kaplan–Meier survival curves for TCGA-LIHC alcohol-HCC patients with respect to the predicted levels of memory $CD8^{+}$ T cells. The red lines indicate the high cell-abundance group, and the green lines indicate the low cell-abundance group.

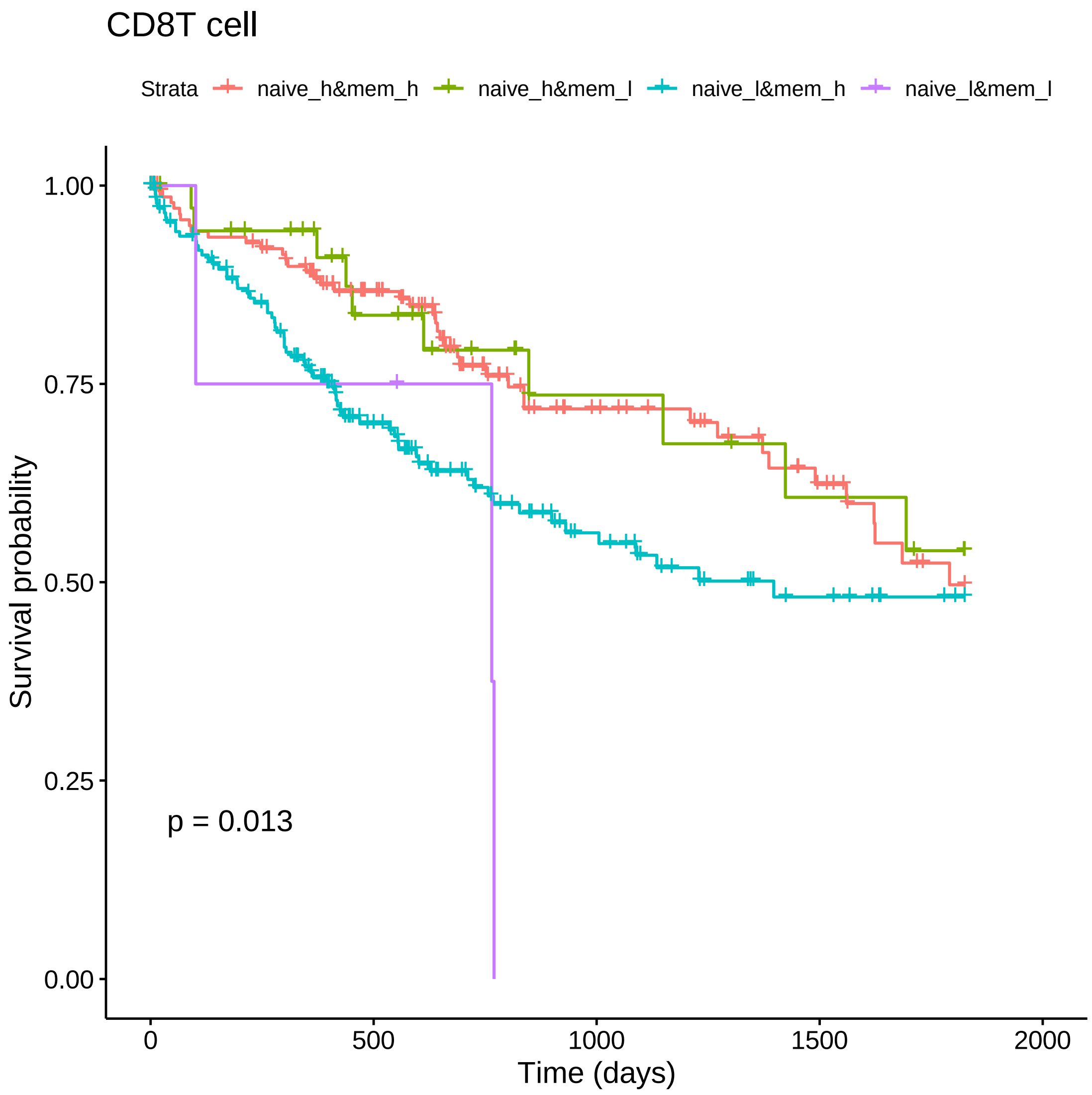

Supplementary Figure 9 Kaplan–Meier survival curves for TCGA-LIHC patients with respect to the predicted levels of naïve CD8^{+} T cells and memory CD8^{+} T cells (higher proportion of naïve CD8^{+} T cells: naïve_h; higher proportion of memory CD8^{+} T cells: mem_h; lower proportion of naïve CD8^{+} T cells: naïve_l; lower proportion of memory CD8^{+} T cells: mem_l)

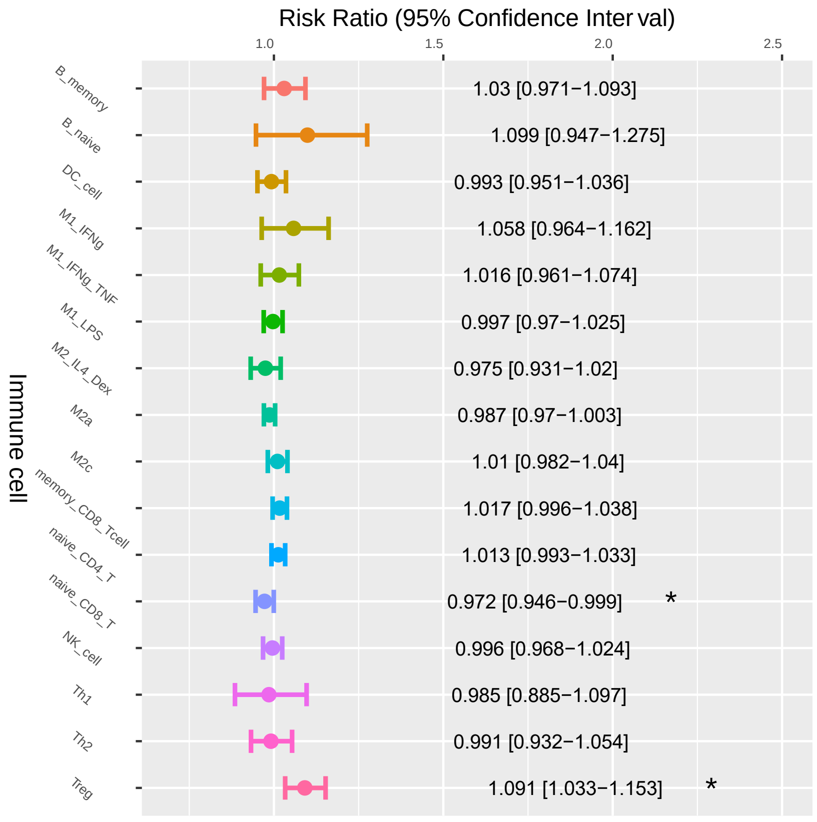

Supplementary Figure 10. Univariate Cox regression analysis between immune cell abundance and the PFS of TCGA-LIHC HCV-HCC patients using the predicted fraction of sixteen immune cell types as the independent variable.

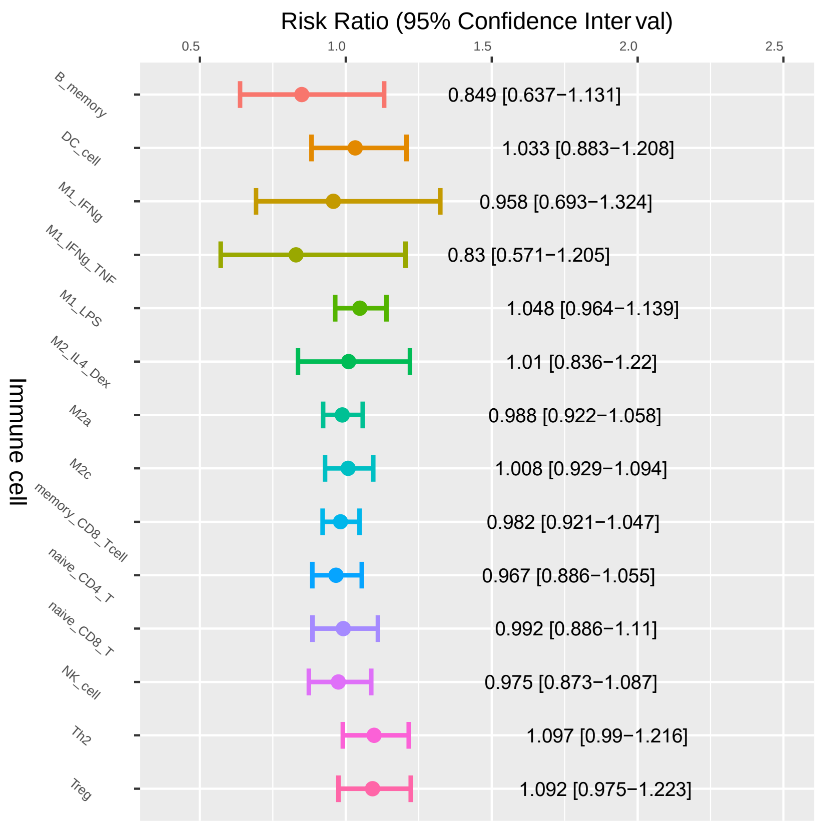

Supplementary Figure 11. Univariate Cox regression analysis between immune cell abundance and the OS of TCGA-LIHC HCV-HCC patients, using the predicted fraction of sixteen immune cell types as the independent variable.

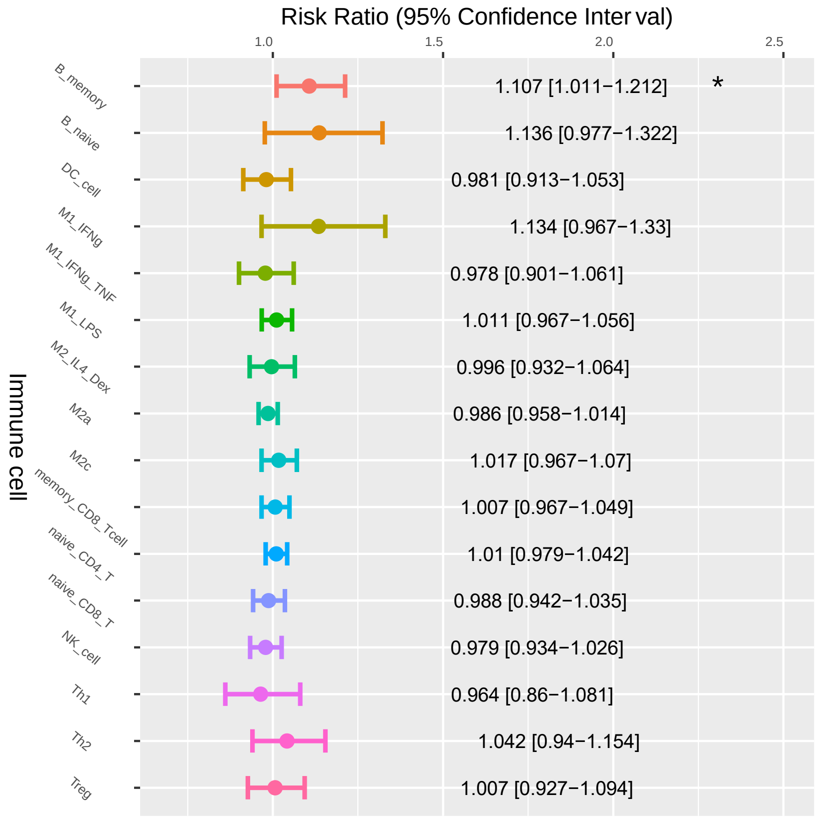

Supplementary Figure 12. Univariate Cox regression analysis between immune cell abundance and the OS of TCGA-LIHC HCC patients without known risk factors, using the fraction of sixteen immune cell types as the independent variable.

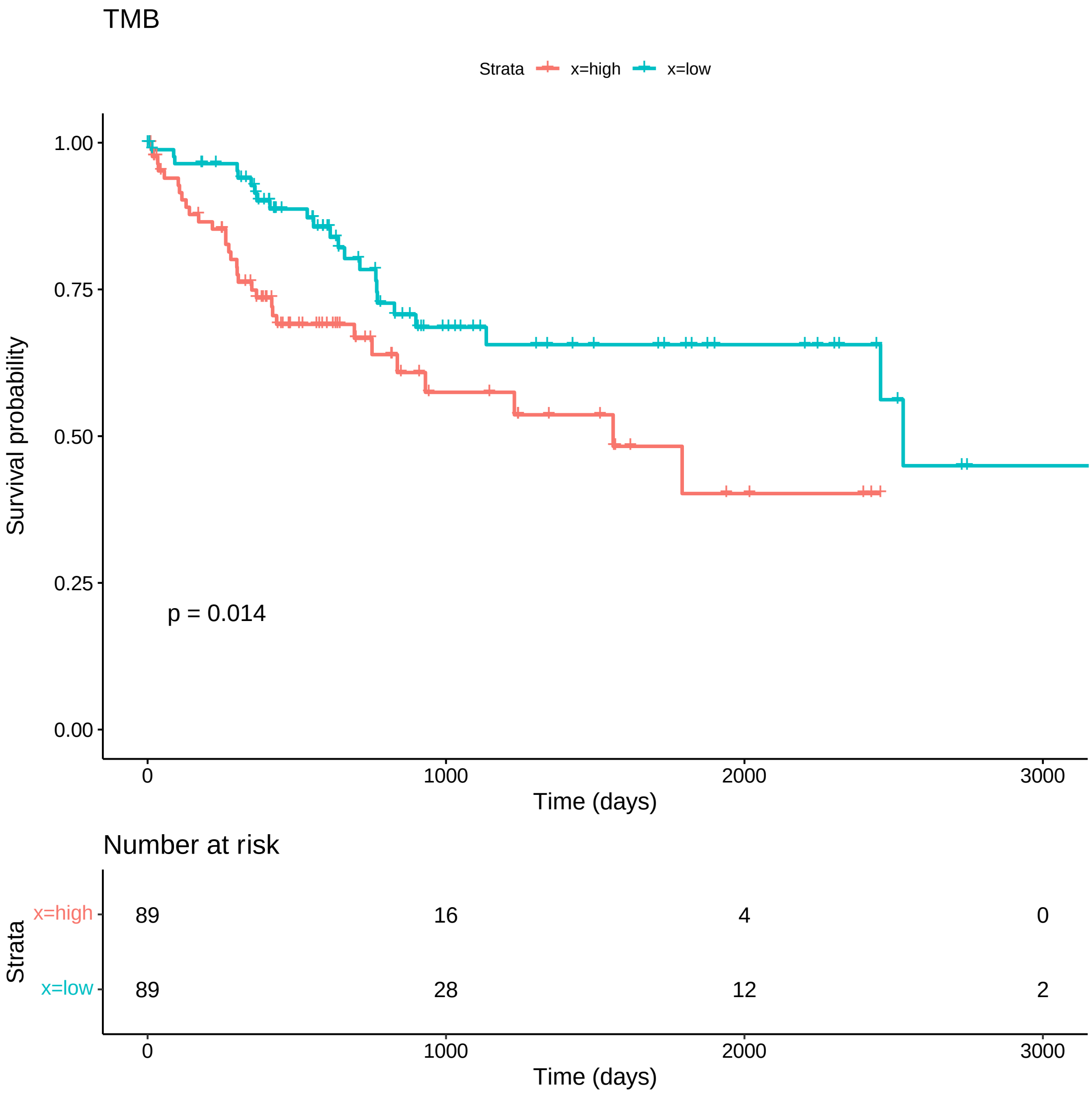

Supplementary Figure 13. Kaplan–Meier survival curves of TCGA-LIHC HCC patients with respect to the levels of tumor mutation burden (TMB). The red line indicates the high TMB group, and the green line indicates the low TMB group.

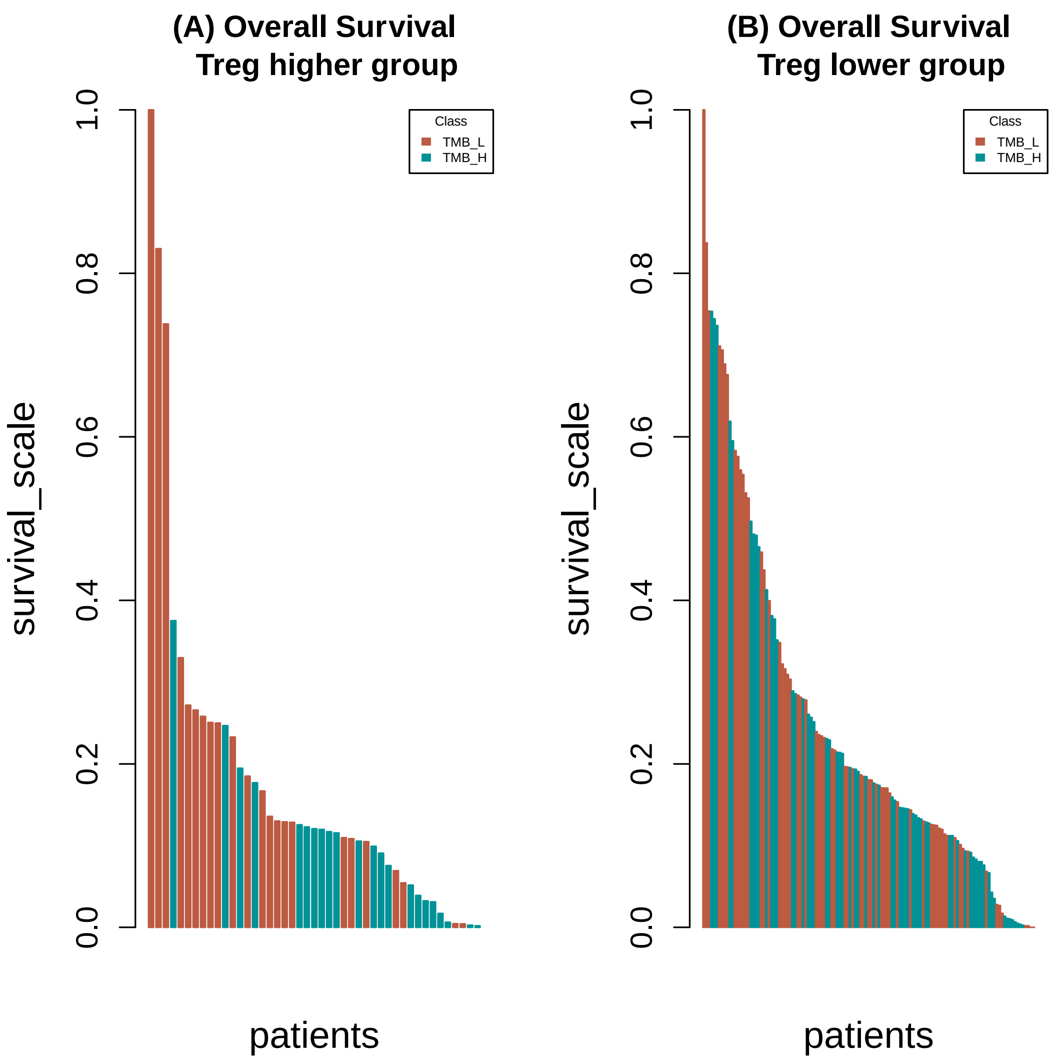

Supplementary Figure 14. Waterfall plot analysis of Treg abundance and TMB on TCGA-LIHC patient survival. The patients are divided into two distinct subgroups, based on HCCImm-predicted Treg abundance, namely the Treg-high and Treg-low groups. The groups are plotted separately to enable a comparative analysis of patient outcomes. The brown bars represent the TMB-high (TMB-h) cases, while the green bars represent the TMB-low (TMB-L) cases. Panel (A) represents the OS for Treg-high patients; (B) represents the OS for Treg-low patients.

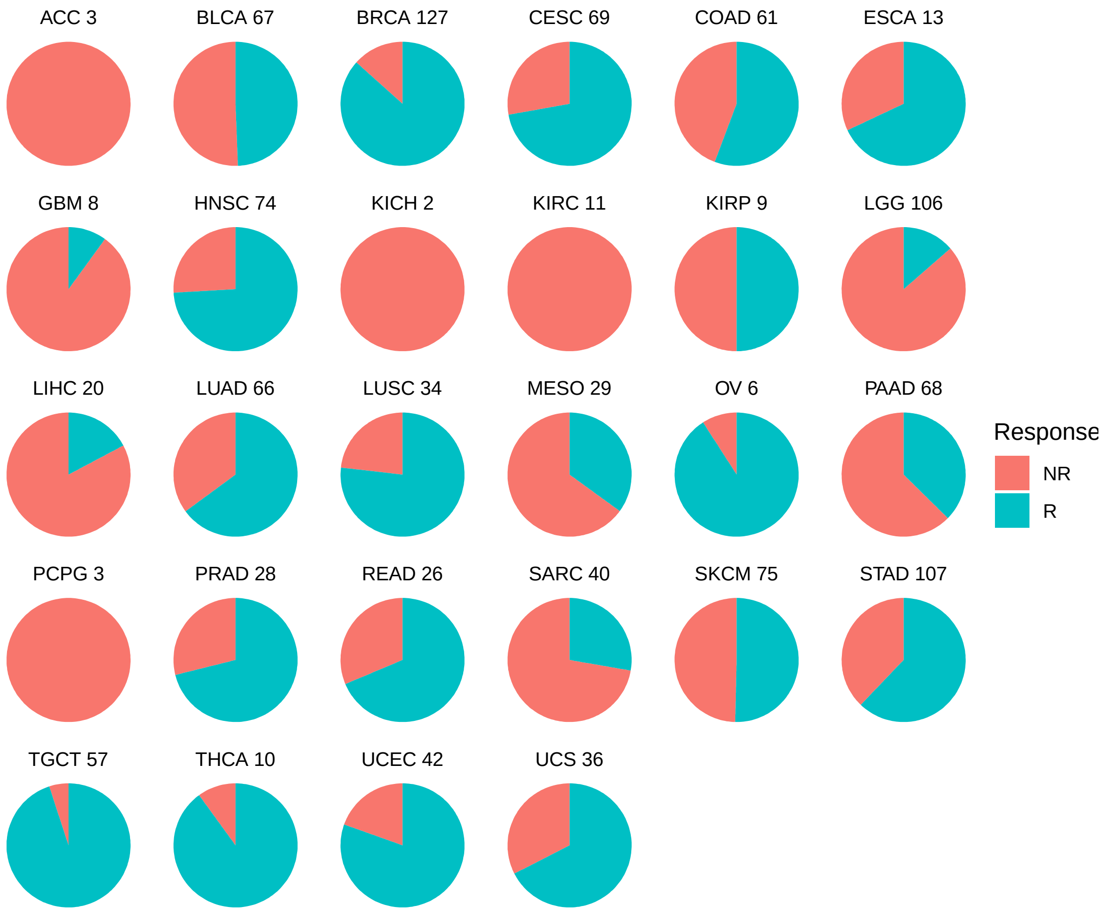

Supplementary Figure 15. Pie charts showing the proportions of cancer patients who had treatment response data in 28 TCGA datasets.

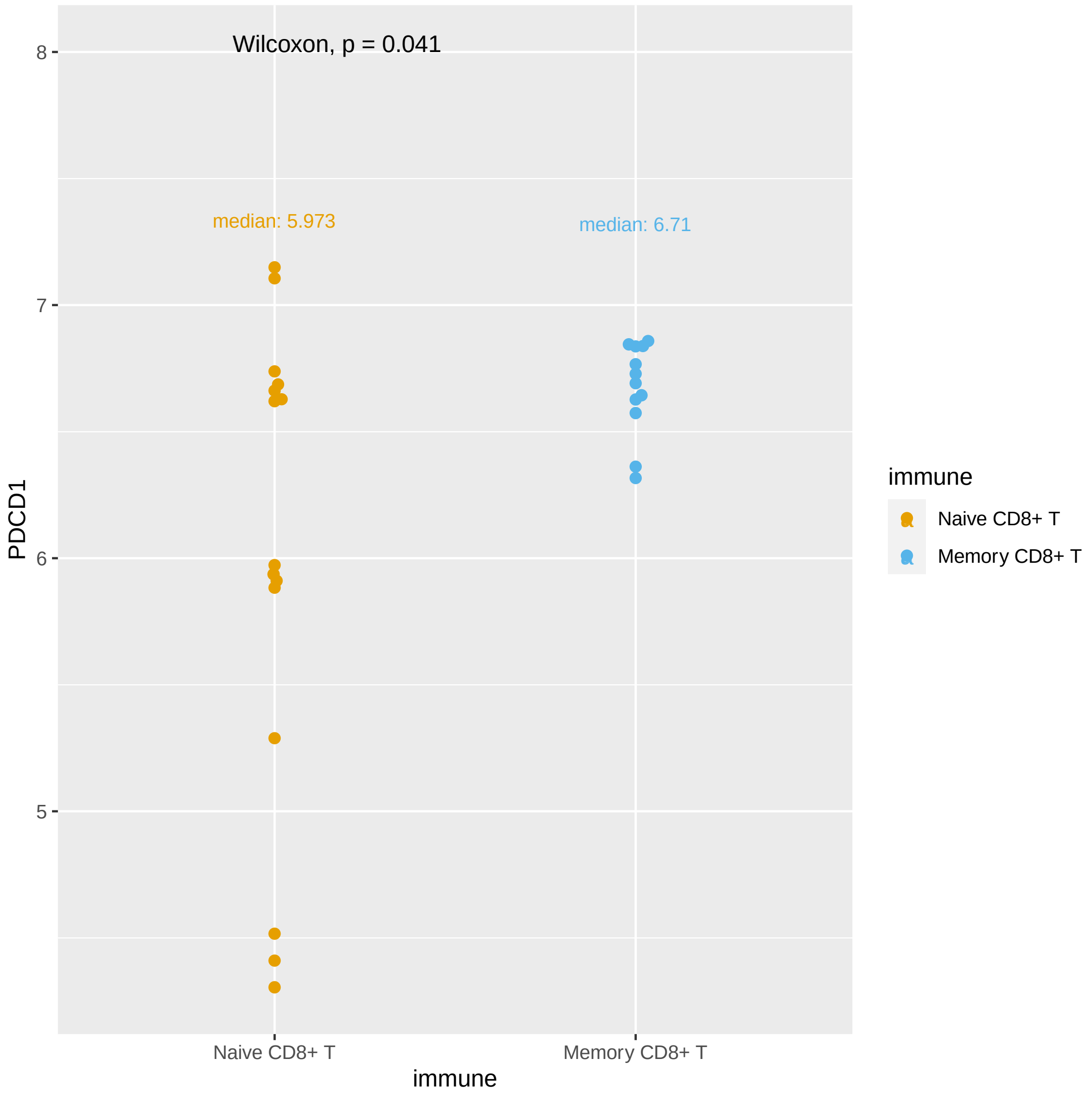

Supplementary Figure 16. Scatter plots revealing the difference in the gene expression levels of PDCD1 between the reference sets of naïve CD8^{+} T cells and memory CD8^{+} T cells. There was a significant difference between the two groups (Wilcoxon signed-rank test, p value < 0.05).

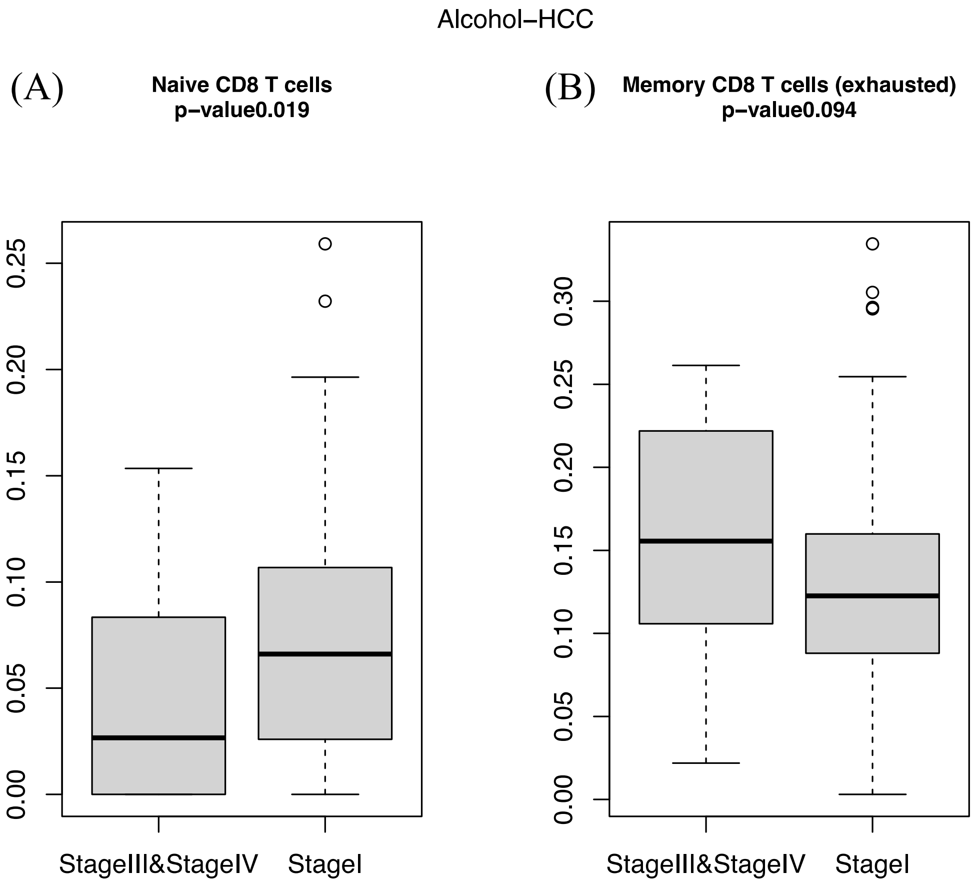

Supplementary Figure 17. Boxplot of the fractions of CD8$^+$ T cells estimated by HCCImm from TCGA-LIHC alcohol-HCC patients. (A) The median fraction of naïve CD8$^+$ T cells in stage III and stage IV was lower than that in stage I. The abundance of naïve CD8$^+$ T cells was significantly different between stage III and stage IV and stage I (t test, p value < 0.05). (B) The median fraction of memory CD8$^+$ T cells in stage III and stage IV was higher than that in stage I. The abundance of memory CD8$^+$ T cells was not significantly different between stage III and stage IV and stage I (t test, p value > 0.05).
